# PLK1/FOXM1-associated tumor-cell state and macrophage-related immune features in endometrial cancer

**DOI:** 10.3389/fonc.2026.1931435

**Published:** 2026-08-26

**Authors:** Longwei Li, Yunyun Xiao, Yaping Wang, Guangyao Song, Ming Liu, Xueling Qu, Lu Han

**Affiliations:** 1 Department of Gynecology, Dalian Women and Children’s Medical Center (Group), The Affiliated Women and Children’s Hospital of Dalian University of Technology, Liaoning Provincial Key Laboratory for Early Diagnosis and Biotherapy of Women’s and Children’s Malignant Tumors, Dalian, China; 2 Graduate School of Dalian Medical University, Dalian, China; 3 Department of Pathology, Dalian Women and Children’s Medical Center (Group), The Affiliated Women and Children’s Hospital of Dalian University of Technology, Liaoning Provincial Key Laboratory for Early Diagnosis and Biotherapy of Women’s and Children’s Malignant Tumors, Dalian, China

**Keywords:** drug sensitivity, endometrial cancer, FoxM1, macrophage, plk1

## Abstract

**Background:**

Polo-like kinase 1 (PLK1) and forkhead box M1 (FOXM1) have been widely studied in various cancers; however, their expression characteristics in endometrial cancer (EC) and their potential association with tumor microenvironment remodeling remain insufficiently characterized.

**Methods:**

This study integrated The Cancer Genome Atlas uterine corpus endometrial carcinoma cohort, Gene Expression Omnibus, pan-cancer transcriptomic data, Human Protein Atlas/Clinical Proteomic Tumor Analysis Consortium, and local immunohistochemistry data to evaluate PLK1 expression and clinicopathological relevance across transcriptomic, proteomic, and histopathological data. Differential expression, survival, gene-set enrichment, transcription-factor enrichment, and immune-infiltration analyses characterized PLK1-associated features. *In vitro* experiments combined EC cell lines AN3CA and HEC-1A with co-immunoprecipitation, Western blotting, Transwell assays, and a THP-1 conditioned-medium model. Drug-response prediction and structure-based analysis prioritized candidate therapeutic hypotheses.

**Results:**

PLK1 was consistently upregulated at both mRNA and protein levels in EC and was associated with higher tumor grade and International Federation of Gynecology and Obstetrics (FIGO) stage. In survival analysis, higher PLK1 expression was associated with poorer overall survival in univariable models but not after adjustment for age, tumor grade, and FIGO stage. Functional enrichment analysis showed that PLK1-associated genes were mainly involved in cell-cycle and mitotic processes. FOXM1 was identified as a potential candidate component of the PLK1-associated transcriptional program and was positively correlated with PLK1 expression and cell-cycle-related features. *In vitro* experiments supported an interaction between PLK1 and FOXM1 and suggested that FOXM1 Thr600 phosphorylation-related alterations were associated with migration and invasion phenotypes. Furthermore, the PLK1/FOXM1-associated tumor-cell state was linked to macrophage-related immune features and changes in the M2-like marker profile of THP-1-derived macrophage-like cells. Drug response analyses suggested differential predicted sensitivity patterns in PLK1-high tumors, providing candidate therapeutic hypotheses for further validation.

**Conclusion:**

The PLK1/FOXM1-associated tumor-cell state may represent a distinct molecular feature associated with proliferative activity, invasive phenotypes, and macrophage-related immune features in EC. This study provides preliminary evidence supporting the biological relevance of this molecular feature and highlights potential therapeutic directions for future investigation.

## Introduction

Endometrial cancer (EC) is one of the most common gynecologic malignancies in women and continues to impose a substantial global disease burden. According to Global Cancer Observatory (GLOBOCAN) 2022 estimates, approximately 420,368 new cases of EC and 97,723 related deaths occurred worldwide, with age-standardized incidence and mortality rates of 8.4 and 1.7 per 100,000, respectively (1). In China, the corresponding numbers were approximately 77,722 new cases and 13,511 deaths (1). Although most patients with early-stage disease achieve favorable outcomes after surgery, prognosis declines markedly with disease progression (2). Population-based data indicate that the 5-year relative survival rate is approximately 96% for localized disease but decreases to about 22% for distant-stage disease (2). In addition, recurrence remains a clinically relevant challenge, with approximately 10%–15% of patients with early-stage EC experiencing relapse, and recurrence risk increasing with advanced stage, aggressive histological subtype, and other adverse clinicopathological features (3). Therefore, further identification of molecular events associated with tumor proliferation, invasion, and immune microenvironment remodeling may help refine risk stratification and provide potential therapeutic targets for high-risk EC (4–6).

Polo-like kinase 1 (PLK1) is a serine/threonine kinase that plays an essential role in cell-cycle regulation, including G2/M transition, centrosome maturation, spindle assembly, chromosome segregation, and mitotic progression. Aberrant PLK1 expression has been reported in multiple malignancies and is frequently associated with increased proliferative activity, aggressive tumor behavior, and unfavorable clinical outcomes, supporting its potential relevance as a therapeutic target (7–11). Forkhead box M1 (FOXM1) is a cell-cycle-associated transcription factor involved in the regulation of cell proliferation, DNA damage response, mitotic progression, and tumor-related transcriptional programs (12–15). Previous studies have shown that PLK1 can interact with and phosphorylate FOXM1, thereby enhancing FOXM1-dependent transcriptional activity; conversely, FOXM1 can also transcriptionally regulate PLK1 and other cell-cycle-related genes, suggesting a positive regulatory network that promotes cell-cycle progression (12, 13).

Tumor-associated macrophages (TAMs) are an important immune component of the EC microenvironment, and M2-like/TAM-related markers such as CD163 and CD206 are commonly used to characterize macrophage-associated immunosuppressive or tumor-promoting states (16, 17). Previous studies have suggested that TAMs may contribute to EC progression through cytokine secretion and immune modulation (17, 18). Recent evidence from other cancer contexts suggests that PLK1/FOXM1-associated programs may participate in tumor invasiveness and macrophage-related immune regulation, providing a rationale for investigating the relationship between the PLK1/FOXM1-associated tumor-cell state and macrophage-associated features in EC (19). However, the role of PLK1/FOXM1-associated tumor-cell–macrophage crosstalk in EC progression remains insufficiently characterized.

Based on this rationale, we integrated The Cancer Genome Atlas uterine corpus endometrial carcinoma (TCGA-UCEC), Gene Expression Omnibus (GEO), Human Protein Atlas/Clinical Proteomic Tumor Analysis Consortium (HPA/CPTAC), pan-cancer expression data, and a local immunohistochemistry (IHC) cohort to characterize PLK1 expression, clinicopathological associations, and functional features in EC. By combining co-immunoprecipitation (Co-IP), Western blotting, Transwell assays, and a THP-1 conditioned-medium model, we further explored whether FOXM1 Thr600 phosphorylation-related alterations are associated with migration and invasion phenotypes and macrophage M2-like marker profiles. Finally, computational drug response prediction and structure-supported analyses were used to prioritize candidate therapeutic hypotheses for PLK1-high EC. These analyses were designed to characterize the PLK1/FOXM1-associated tumor-cell state and its relationship with macrophage-related immune features in EC progression.

## Materials and methods

### Data sources and preprocessing

A local clinical validation cohort was established using formalin-fixed paraffin-embedded endometrial tissue samples collected at Dalian Women and Children’s Medical Center (Group) between January 2022 and December 2024. The cohort included 54 EC tissues and 10 normal endometrial tissues. Eligible EC cases were pathologically confirmed primary endometrial carcinomas with available tissue specimens and complete clinicopathological information. Patients who had received preoperative antitumor treatment, had concurrent malignancies, recurrent EC, metastatic tumors involving the endometrium, poor-quality tissue specimens, or missing key clinical data were excluded. Normal endometrial control tissues were obtained from 10 patients with cervical cancer undergoing surgery and were pathologically confirmed to be free of endometrial malignancy or atypical hyperplasia. This study was approved by the Ethics Committee of Dalian Women and Children’s Medical Center (Group) (approval No. KY-2025-97).

The TCGA-UCEC RNA-sequencing (RNA-seq) HTSeq expression matrix, reported as fragments per kilobase of transcript per million mapped reads (FPKM), and corresponding clinical data were obtained from the Genomic Data Commons (GDC) Data Portal and interpreted in the context of the TCGA endometrial carcinoma molecular characterization study (20). The data were used to evaluate PLK1 and FOXM1 expression, clinicopathological associations, pathway activity, immune microenvironment features, and overall survival. Expression values were transformed as log2(FPKM + 1). Primary tumor and solid tissue normal samples were distinguished according to TCGA barcode annotations. Samples with defined tissue type and available target-gene expression values were retained, whereas samples with unknown type, missing expression values, or duplicated sequencing records were excluded. A total of 551 tumor samples and 23 normal samples were included for expression analysis. Clinicopathological analyses were performed at the patient level after removing duplicated records. For overall survival analysis, cases with missing survival status, missing follow-up time, or follow-up time <30 days were excluded, leaving 510 eligible cases. For Kaplan–Meier analyses, PLK1-high/low and FOXM1-high/low groups were redefined using the median expression of the corresponding gene within the eligible survival cohort; cohort-specific medians were used for other grouped analyses. For the local IHC cohort, PLK1 IHC-high/low groups were defined according to the median PLK1 IHC score among tumor samples. Median-based stratification was selected to provide a consistent and reproducible approach for defining high- and low-expression states across transcriptomic datasets and the local tissue cohort, thereby facilitating comparable downstream analyses of molecular features, immune contexture, and functional phenotypes. Since the primary objective of this study was to characterize the PLK1/FOXM1-associated tumor-cell state rather than to develop a prognostic classification model, survival-optimized cutoffs were not adopted as the primary grouping strategy. The external validation dataset GSE17025 was downloaded from the Gene Expression Omnibus database (21). This dataset was generated on the GPL570 platform (Affymetrix Human Genome U133 Plus 2.0 Array). After platform annotation, PLK1 and FOXM1 expression values were extracted from 91 EC tissues and 12 normal endometrial tissues to validate tumor-normal expression differences. Pan-cancer expression analyses were performed using the uniformly processed TCGA/Therapeutically Applicable Research to Generate Effective Treatments (TARGET)/Genotype-Tissue Expression (GTEx) RNA-seq matrix from the University of California, Santa Cruz (UCSC) Xena platform (22). Tumor and normal sample annotations were matched using the corresponding phenotype files. PLK1 protein expression, CPTAC proteomic differences, and representative immunohistochemistry images were retrieved from the Human Protein Atlas PLK1 entry and cancer atlas resources (23).

### Functional enrichment and immune microenvironment analyses

In the TCGA-UCEC cohort, Spearman rank correlation analysis was performed between PLK1 expression and the whole-transcriptome expression profile. Genes were ranked by their correlation coefficients, and gene set enrichment analysis (GSEA) was conducted using Kyoto Encyclopedia of Genes and Genomes (KEGG) and Hallmark gene sets from the Molecular Signatures Database (MSigDB) (24, 25). Pathways of interest included cell cycle, DNA replication, E2F targets, G2/M checkpoint, mitotic spindle, MYC targets, DNA repair, and epithelial-mesenchymal transition (EMT)-related signatures. Genes upregulated in PLK1-high tumors were further submitted to Enrichr transcription-factor target enrichment libraries, with emphasis on FOXM1, E2F family members, and MYBL2-related transcriptional regulatory programs (26). FOXM1 was then analyzed as a candidate associated molecular feature. The correlation between PLK1 and FOXM1 expression was calculated, and FOXM1 expression, FOXM1 co-expression score, E2F targets, G2/M checkpoint, mitotic spindle, and proliferation scores were compared between PLK1-high and PLK1-low samples. Hallmark pathway scores were also compared between PLK1-high/low and FOXM1-high/low groups to identify pathways commonly increased in both high-expression states.

The immune microenvironment was evaluated using CIBERSORT R script v1.03 with the LM22 leukocyte signature matrix (22 immune-cell subsets) and 1,000 permutations (27). Quantile normalization was disabled for the bulk RNA-seq input. Estimation of STromal and Immune cells in MAlignant Tumor tissues using Expression data (ESTIMATE) was used to calculate ImmuneScore (28). Predefined marker gene sets were used to calculate z scores for pan-macrophage, M1-like macrophage, and M2-like TAM signatures. Associations with PLK1/FOXM1 expression were assessed using two-sided Spearman rank correlation analysis.

### Immunohistochemistry

Formalin-fixed paraffin-embedded tissue sections from the local validation cohort were used for immunohistochemical staining. After deparaffinization, rehydration, antigen retrieval, and endogenous peroxidase blocking, sections were incubated with the following primary antibodies: anti-PLK1 rabbit monoclonal antibody (mAb) (208G4, 4513S/4513T), anti-FOXM1 rabbit mAb (D3F2B, 20459T), anti-CD86 rabbit mAb (E2G8P, 91882S), anti-CD163 rabbit mAb (D6U1J, 93498T), and anti-CD206/MRC1 rabbit mAb (E2L9N, 91992T/91992S). All primary antibodies were purchased from Shanghai Universal Biotech Co., Ltd. Detection was performed using SignalStain^®^ Boost IHC Detection Reagent (horseradish peroxidase [HRP], Rabbit; Cell Signaling Technology, 8114/8114S; purchased from Shanghai Universal Biotech Co., Ltd.), followed by visualization with 3,3′-diaminobenzidine (DAB) using the SignalStain^®^ DAB Substrate Kit (Cell Signaling Technology, 8059/8059P or 8059S; purchased from Shanghai Universal Biotech Co., Ltd.). Sections were counterstained with hematoxylin, dehydrated, mounted, and examined microscopically. All staining procedures were performed according to the manufacturers’ protocols. All slides were independently evaluated by two observers using unified criteria. Each slide was first reviewed at low magnification to assess overall staining and to avoid necrotic areas, hemorrhagic areas, vascular-dense regions, and tissue-folding artifacts. For each case, five non-overlapping high-power fields (400×) were randomly selected within the tumor region, and marker-positive cells were manually counted. Cells with clear membranous and/or cytoplasmic brown-yellow to brown staining were considered positive, whereas nonspecific staining was excluded. The average number of positive cells across the five high-power fields was used as the positive-cell density for each case. A 0–3 semiquantitative density score was assigned as follows: 0 for ≤5 positive cells per high-power field, 1 for >5 to ≤20, 2 for >20 to ≤50, and 3 for >50. Discrepant evaluations were resolved by joint review. Based on the median PLK1 IHC score among the 54 EC samples, cases were divided into PLK1 IHC-high and PLK1 IHC-low groups.

### Cell models, transfection, and treatment groups

Human EC cell lines AN3CA and HEC-1A, as well as the human monocytic cell line THP-1, were used for *in vitro* experiments. AN3CA, HEC-1A, and THP-1 cells were obtained from Procell/Pricella (Wuhan, China; CL-0505, CL-0099, and CL-0233, respectively). AN3CA cells were cultured in minimum essential medium (MEM), HEC-1A cells in McCoy’s 5A medium, and THP-1 cells in Roswell Park Memorial Institute 1640 (RPMI-1640) medium. All media were supplemented with 10% fetal bovine serum and 1% penicillin–streptomycin; THP-1 medium was additionally supplemented with 0.05 mmol/L β-mercaptoethanol. Cells were maintained at 37 °C in a humidified incubator containing 5% CO_2_ and were confirmed to be mycoplasma-negative before experiments. PLK1 knockdown and overexpression models were generated using small interfering RNA/short hairpin RNA (siRNA/shRNA) and overexpression plasmids or lentiviral vectors, respectively. PLK1 siRNA/shRNA and corresponding negative controls were synthesized by Shanghai GenePharma Co., Ltd.; plasmid or lentiviral vectors were also constructed by Shanghai GenePharma Co., Ltd. Transient transfection was performed using Lipofectamine RNAiMAX or Lipofectamine 3000 (Invitrogen/Thermo Fisher Scientific) according to the manufacturer’s instructions. Cells were harvested 24–48 h after transfection or infection, and PLK1 knockdown, PLK1 overexpression, and FOXM1 mutant expression were verified by quantitative reverse-transcription polymerase chain reaction (qRT-PCR) and/or Western blotting. FOXM1-T600A was generated by substituting threonine 600 of FOXM1 with alanine and was constructed as a pCDNA3.1-CMV-V5-FOXM1-T600A-3*FLAG expression vector; the corresponding wild-type FOXM1 (FOXM1-WT) vector was used as the control. The vector records were E-1146 for FOXM1-WT and E-1147 for FOXM1-T600A. This mutant, also referred to as FOXM1-A in experimental group labels, was used to evaluate the functional relevance of FOXM1 Thr600 phosphorylation deficiency in migration and invasion and macrophage-related phenotypes. The PLK1 inhibitor BI 2536 (Selleck Chemicals, S1109) and the FOXM1 inhibitor FDI-6 (Selleck Chemicals, S9689) were dissolved in dimethyl sulfoxide (DMSO) and diluted in complete culture medium before treatment. BI 2536 and FDI-6 were used at final concentrations of 30 nM and 15 nM, respectively, for 24 h before subsequent molecular and functional assays. The final DMSO concentration was maintained at 0.1% (volume/volume [v/v]) in all treatment groups, and control cells received an equal volume of DMSO as the vehicle control. Experimental groups included control (Ctrl), PLK1 knockdown (PLK1-KD), PLK1 overexpression (PLK1-OE), FOXM1-T600A, PLK1 inhibitor (PLK1-i; BI 2536, 30 nM for 24 h), and FOXM1 inhibitor (FOXM1-i; FDI-6, 15 nM for 24 h). Ctrl groups received negative-control siRNA/shRNA, empty vector, or 0.1% DMSO vehicle treatment, as appropriate.

### qRT-PCR, Western blotting, and Co-IP

PLK1 transfection efficiency and macrophage polarization-related gene expression were examined by qRT-PCR. Total RNA was extracted using TRIzol(TM) Reagent (Invitrogen/Thermo Fisher Scientific, 15596026), and reverse transcription was performed using HiScript III RT SuperMix for qPCR (Vazyme, R323-01). The amplification reaction was performed using ChamQ Universal SYBR qPCR Master Mix (Vazyme, Q711-02/Q711-03), with glyceraldehyde-3-phosphate dehydrogenase (GAPDH) as the internal control. The M1-like markers included NOS2, IL12A, and TNF, and the M2-like markers included ARG1, IL10, and TGFB1. Relative expression was calculated using the 2^−ΔΔCt method. Primers were synthesized by Sangon Biotech (Shanghai, China), and primer sequences are listed in **Supplementary Table 1**. Western blotting was used to detect PLK1, FOXM1, and phosphorylated FOXM1 at Thr600 [p-FOXM1(Thr600)]. Total cellular protein was extracted using radioimmunoprecipitation assay (RIPA) lysis buffer (Beyotime, P0013B), and protein concentrations were determined using a bicinchoninic acid (BCA) Protein Assay Kit (Beyotime, P0012). Equal amounts of protein were separated by sodium dodecyl sulfate–polyacrylamide gel electrophoresis (SDS-PAGE) and transferred onto polyvinylidene difluoride (PVDF) membranes (Merck Millipore, IPVH00010). After blocking, membranes were incubated with primary antibodies, followed by HRP-conjugated secondary antibodies and chemiluminescent detection. Primary antibodies included PLK1 (Cell Signaling Technology [CST], 4513S/4513T), FOXM1 (CST, 20459S/20459T), phospho-FOXM1(Thr600) (CST, 14655), and GAPDH (CST, 5174S/5174T). Western blot band intensities were quantified using Fiji (ImageJ v1.54t; National Institutes of Health, Bethesda, MD, USA). Band integrated density was measured from nonsaturated images acquired under identical exposure conditions using consistent regions of interest. Local background was subtracted before densitometry, and no binary intensity threshold was applied. Total protein levels were normalized to GAPDH, whereas p-FOXM1(Thr600) was normalized to total FOXM1 from the corresponding lane; relative values were expressed against the Ctrl group. Because FOXM1-T600A abolishes the Thr600 epitope recognized by the phospho-specific antibody, its reduced p-FOXM1(Thr600) signal was not considered independent evidence of altered phosphorylation. Co-IP was performed to assess the association between PLK1 and FOXM1. Cells were transfected with FOXM1-WT-V5 or FOXM1-T600A-V5 plasmids. Protein lysates were collected, and input, immunoglobulin G (IgG) negative control, and anti-V5 immunoprecipitation groups were established. V5-tagged FOXM1 was immunoprecipitated using anti-V5 antibody, and co-precipitated PLK1 was detected by Western blotting. Co-IP was used to provide evidence of PLK1–FOXM1 association and was not interpreted as a comparison of binding affinity between FOXM1-WT and FOXM1-T600A.

### Transwell migration and invasion assays

Transwell assays were used to assess migration and invasion of EC cells. AN3CA cells were divided into six groups: Ctrl, PLK1-KD, PLK1-OE, FOXM1-T600A, PLK1-i, and FOXM1-i. HEC-1A cells were divided into four groups: Ctrl, PLK1-KD, PLK1-OE, and FOXM1-T600A. For transfection experiments, cells were seeded into six-well plates at 4 × 10^5 cells/well and treated according to the corresponding transfection protocols after attachment. For migration assays, 24-well Transwell inserts with 8.0 μm pores (Corning, 3422) were used. Treated cells were counted, resuspended in serum-free medium, and equal numbers of viable cells from each group were seeded into the upper chamber. The lower chamber was filled with 600 μL complete medium. For invasion assays, the upper chamber was precoated with Matrigel Basement Membrane Matrix (Corning, 356234). According to the available experimental record, Matrigel was diluted 1:8, 50 μL was added to each insert, and the inserts were incubated at 37 °C for 30 min to allow gelation; equal numbers of viable cells were then seeded into the upper chamber. After 48 h, non-migrated or non-invaded cells on the upper membrane surface were removed. Cells on the lower membrane surface were fixed with 4% paraformaldehyde (Beyotime, P0099) for 20 min and stained with crystal violet solution (Beyotime, C0121) for 30 min. Images were captured under a microscope, and cell numbers were quantified using ImageJ.

### Conditioned-medium-induced THP-1 polarization model

An indirect conditioned-medium model was used to evaluate the effect of the PLK1/FOXM1-associated tumor-cell state on macrophage-like polarization phenotypes. THP-1 cells were treated with 50 ng/mL phorbol 12-myristate 13-acetate (PMA; Sigma-Aldrich, P8139) for 24 h to induce adherent macrophage-like differentiation, followed by replacement with fresh medium and recovery for 24 h. Conditioned medium was prepared from AN3CA or HEC-1A cells from different treatment groups. After transfection or 24-h inhibitor treatment with BI 2536 (30 nM) or FDI-6 (15 nM), tumor cells at approximately 70%–80% confluence were washed and cultured in low-serum medium for a further 24 h to generate conditioned medium. During inhibitor treatment, the final DMSO concentration was maintained at 0.1% (v/v), and the corresponding control cells received an equal volume of DMSO. Supernatants were collected, centrifuged at 300 × g for 5 min to remove cellular debris, filtered through a 0.22 μm filter, and used as conditioned medium. PMA-differentiated THP-1 cells were then treated with 100% conditioned medium for 48 h. The AN3CA conditioned-medium experiment included Ctrl, PLK1-KD, PLK1-OE, FOXM1-T600A, PLK1-i, and FOXM1-i groups, whereas the HEC-1A conditioned-medium experiment included Ctrl, PLK1-KD, PLK1-OE, and FOXM1-T600A groups. This model was an indirect conditioned-medium system and did not involve direct contact co-culture between tumor cells and THP-1-derived cells.

### Flow cytometry and enzyme-linked immunosorbent assay

After conditioned-medium treatment, THP-1-derived cells were collected for flow cytometry, and culture supernatants were collected for ELISA. Cellular RNA was also extracted for polarization-related qRT-PCR analysis. Flow cytometry was used to assess CD86 and CD163 expression. Cells were washed with phosphate-buffered saline (PBS), blocked with 3% bovine serum albumin (BSA) at 4 °C for 1.5 h, and adjusted to approximately 1 × 10^6^ cells per 100 μL cell suspension. Cells were then incubated with fluorescein isothiocyanate (FITC)-conjugated anti-human CD86 antibody (BioLegend, clone BU63, 374203/374204) and phycoerythrin (PE)-conjugated anti-human CD163 antibody (BioLegend, clone GHI/61, 333605/333606) at 4 °C for 30 min in the dark. After washing, cells were resuspended and analyzed using a NovoCyte 2040R flow cytometer. Single-stained controls were included for compensation. During analysis, cellular debris was excluded using forward-scatter/side-scatter (FSC/SSC) gating, followed by singlet gating; CD86^+^ and CD163^+^ proportions were analyzed within the target cell population. Polarization-related gene expression was assessed using the qRT-PCR method described above. The measured markers included NOS2, IL12A, and TNF for M1-like features and ARG1, IL10, and TGFB1 for M2-like features. ELISA was used to quantify interleukin (IL)-12p70, tumor necrosis factor (TNF)-α, IL-10, and transforming growth factor (TGF)-β1 in culture supernatants using Human IL-12p70 ELISA Kit (MultiSciences, EK112), Human TNF-α ELISA Kit (MultiSciences, EK182), Human IL-10 ELISA Kit (MultiSciences, EK110), and Human/Mouse/Rat TGF-β1 ELISA Kit (MultiSciences, EK981), respectively. Standard curves were generated, and sample concentrations were calculated according to the manufacturers’ instructions.

### Computational drug prioritization and structure-supported analysis

Based on the TCGA-UCEC RNA-seq expression matrix, samples were divided into PLK1-high and PLK1-low groups according to median PLK1 expression. Differential expression analysis was performed, and genes meeting an absolute log2 fold change (|log2FC|) > 1 and a false discovery rate (FDR) < 0.05 were used to construct PLK1-high-associated upregulated and downregulated gene lists. These gene lists were submitted to the Library of Integrated Network-Based Cellular Signatures (LINCS) L1000 Characteristic Direction Signature Search Engine (L1000CDS2) and Enrichr perturbation resources to identify small molecules predicted to reverse the PLK1-high transcriptional signature (26, 29). Candidate drug responses were further predicted using the R package oncoPredict in the TCGA-UCEC expression matrix (30). Predicted drug response metrics were compared between PLK1-high and PLK1-low groups using the Wilcoxon rank-sum test, and multiple comparisons were adjusted using the Benjamini-Hochberg method.

Structure-supported analysis of PLK1 inhibitors was performed using the PLK1-BI 2536 co-crystal structure 2RKU and the PLK1 surface-entropy-reduction mutant complexed with GSK461364 structure 9R1Y from the Research Collaboratory for Structural Bioinformatics (RCSB) Protein Data Bank. Structural files were downloaded and analyzed using PyMOL and/or the Protein–Ligand Interaction Profiler (PLIP) (31). Residues within 4 Å of the ligand were identified, and potential hydrogen bonds, hydrophobic interactions, and other non-covalent interactions were recorded.

### Statistical analysis

Continuous variables were analyzed using appropriate statistical tests according to data distribution and the number of comparison groups. Two-group comparisons were performed using the Mann–Whitney U test or Welch’s t-test as appropriate. Paired comparisons were performed using the paired t-test or Wilcoxon signed-rank test. Multiple-group comparisons were performed using the Kruskal–Wallis test. Categorical variables were compared using the χ² test or Fisher’s exact test. Correlations were assessed using two-sided Spearman rank correlation analysis based on patient-level deduplicated data and pairwise complete observations. Samples missing either variable were excluded from the corresponding comparison. For multiple testing, *P* values were adjusted using the Benjamini–Hochberg method, with FDR reported where applicable.

Overall survival curves were generated using the Kaplan–Meier method, and between-group differences were assessed using the log-rank test. Cox proportional hazards regression was used to evaluate the association between PLK1 and overall survival, with hazard ratios (HRs) and 95% confidence intervals (CIs) reported. The primary model included z-standardized PLK1 (HR per 1-standard deviation [SD] increase), age (per 10-year increase), tumor grade (G3 vs. G1–G2), and International Federation of Gynecology and Obstetrics (FIGO) stage (III–IV vs. I–II). These covariates were prespecified based on clinical relevance and data availability; no automated stepwise selection was used. A sensitivity model replaced continuous PLK1 with median-defined PLK1-high versus PLK1-low status while retaining age, grade, and stage. The proportional hazards assumption was assessed using Schoenfeld residuals. Time-dependent receiver operating characteristic (ROC) curves and corresponding areas under the curve (AUCs) were used to evaluate the discriminatory ability of PLK1 for 1-, 3-, and 5-year overall survival.

*In vitro* experiments were performed with at least three independent biological replicates, and data are presented as mean ± SD. All statistical tests were two-sided, and *P* < 0.05 was considered statistically significant. Statistical analyses and visualization were performed using Python and R. The main Python packages included pandas, numpy, scipy, statsmodels, lifelines, matplotlib, seaborn, openpyxl, and Biopython. The main R packages included survival, timeROC, oncoPredict, data.table, and ggplot2. CIBERSORT immune infiltration analysis was performed using CIBERSORT R script v1.03, and drug-response prediction was performed using oncoPredict.

## Results

### PLK1 expression and clinicopathological associations

**Figure 1:**
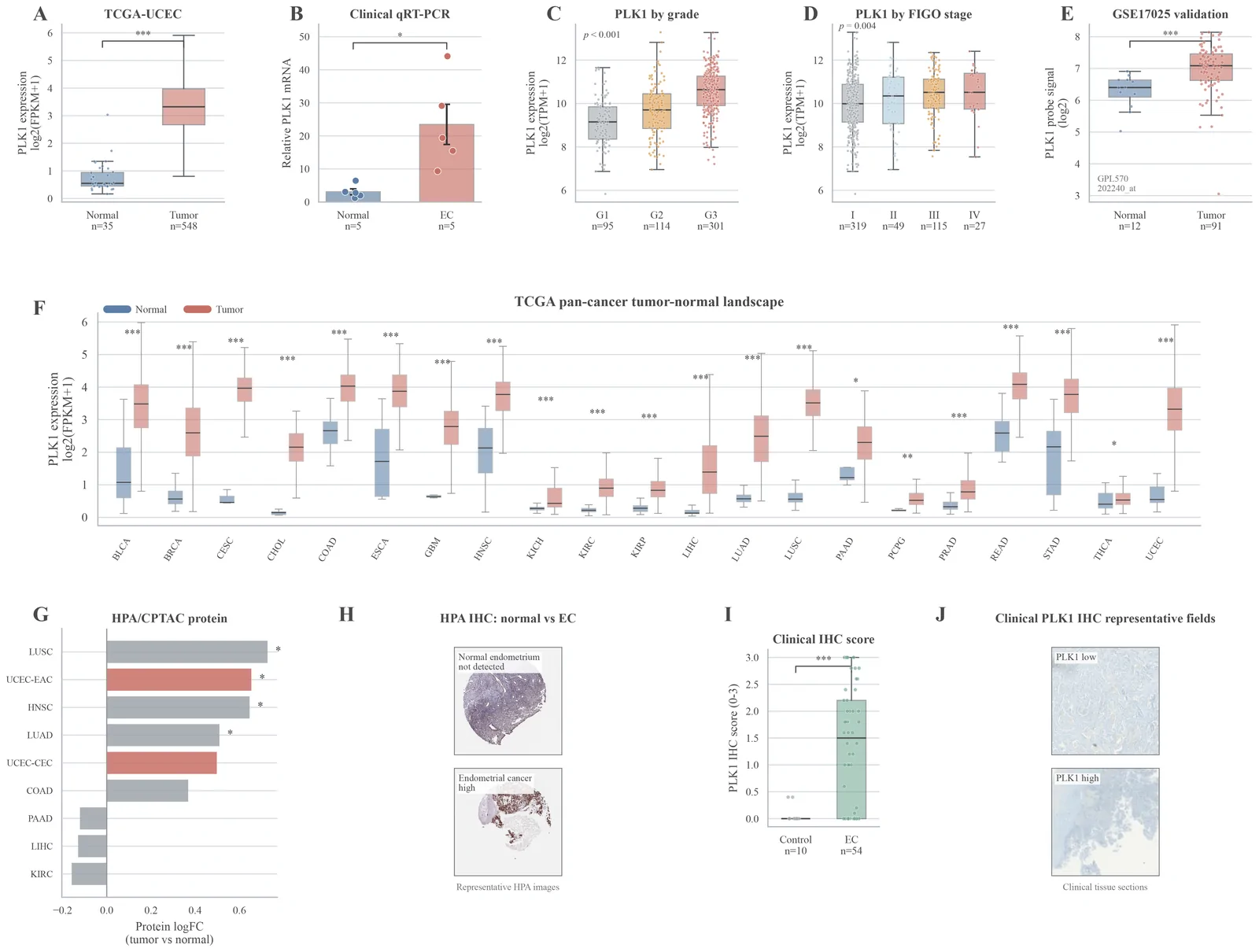
PLK1 is upregulated in EC and associated with aggressive clinicopathological features. **(A)** PLK1 expression in TCGA-UCEC tumor and normal endometrial tissues. **(B)** Local paired qRT-PCR validation of PLK1 mRNA expression. **(C, D)** Association of PLK1 expression with tumor grade and FIGO stage in TCGA-UCEC. **(E)** External validation of PLK1 expression in GSE17025. **(F)** Pan-cancer tumor-normal expression profile of PLK1. **(G)** HPA/CPTAC protein-level evidence for PLK1 expression across cancers. **(H)** Representative HPA IHC images of PLK1 in normal endometrium and EC. **(I)** Local PLK1 IHC score comparison between normal and tumor tissues. **(J)** Representative local PLK1 IHC staining images. PLK1, polo-like kinase 1; EC, endometrial cancer; TCGA, The Cancer Genome Atlas; UCEC, uterine corpus endometrial carcinoma; FPKM, fragments per kilobase of transcript per million mapped reads; TPM, transcripts per million; qPCR, quantitative polymerase chain reaction; qRT-PCR, quantitative reverse-transcription polymerase chain reaction; mRNA, messenger RNA; FIGO, International Federation of Gynecology and Obstetrics; G1–G3, histological grades 1–3; GEO, Gene Expression Omnibus; GSE17025, GEO Series accession GSE17025; GPL570, Affymetrix Human Genome U133 Plus 2.0 Array platform; 202240_at, Affymetrix probe-set identifier for PLK1; HPA, Human Protein Atlas; CPTAC, Clinical Proteomic Tumor Analysis Consortium; IHC, immunohistochemistry; logFC, log2 fold change; n, number of samples. TCGA cancer-type abbreviations are as follows: BLCA, bladder urothelial carcinoma; BRCA, breast invasive carcinoma; CESC, cervical squamous cell carcinoma and endocervical adenocarcinoma; CHOL, cholangiocarcinoma; COAD, colon adenocarcinoma; ESCA, esophageal carcinoma; GBM, glioblastoma multiforme; HNSC, head and neck squamous cell carcinoma; KICH, kidney chromophobe; KIRC, kidney renal clear cell carcinoma; KIRP, kidney renal papillary cell carcinoma; LIHC, liver hepatocellular carcinoma; LUAD, lung adenocarcinoma; LUSC, lung squamous cell carcinoma; PAAD, pancreatic adenocarcinoma; PCPG, pheochromocytoma and paraganglioma; PRAD, prostate adenocarcinoma; READ, rectum adenocarcinoma; STAD, stomach adenocarcinoma; THCA, thyroid carcinoma. UCEC-EAC, uterine endometrioid adenocarcinoma; UCEC-CEC, uterine clear cell carcinoma. *P < 0.05, **P < 0.01 and ***P < 0.001.

In the TCGA-UCEC cohort, PLK1 expression was higher in EC tissues than in normal endometrial tissues (*P* < 0.001; **Figure 1A**). A similar trend was observed in the local qRT-PCR analysis of five paired tumor and adjacent normal tissue samples (*P* < 0.05; **Figure 1B**). Clinicopathological stratification showed that PLK1 expression differed across tumor grades and FIGO stages, and was moderately or weakly positively correlated with tumor grade and FIGO stage, respectively (**Figures 1C, D**). The TCGA-UCEC clinicopathological characteristics stratified by PLK1 expression are summarized in **Table 1**. External validation further supported these findings. In the GSE17025 dataset, PLK1 expression was higher in EC tissues than in normal endometrial tissues (*P* < 0.001; **Figure 1E**). Pan-cancer analysis also suggested elevated PLK1 expression in multiple tumor types, including UCEC (**Figure 1F**). HPA/CPTAC proteomic and immunohistochemical data indicated higher PLK1 protein expression and staining intensity in EC tissues than in normal tissues (**Figures 1G, H**). Consistently, in the local IHC cohort, PLK1 scores were higher in 54 EC tissues than in 10 normal endometrial controls (*P* < 0.001; **Figure 1I**), and representative PLK1-positive and PLK1-negative staining images are shown in **Figure 1J**. Overall, these results suggest that PLK1 is relatively highly expressed in EC and may be associated with higher tumor grade and stage.

**Table 1:** TCGA-UCEC clinicopathological characteristics stratified by PLK1 expression.

Characteristic	Overall	PLK1-low	PLK1-high	*P* value
Cases, n	530	265	265	
Clinical follow-up				
Age, years, median [IQR]	64.00 [57.00-71.00]	63.00 [56.00-71.00]	64.00 [57.00-71.00]	0.309
Follow-up time, days, median [IQR]	897.50 [512.25-1566.50]	943.00 [566.00-1817.00]	824.00 [455.00-1407.00]	0.007
Overall survival status, n/N (%)				0.114
Censored	450/530 (84.9%)	232/265 (87.5%)	218/265 (82.3%)	
Death	80/530 (15.1%)	33/265 (12.5%)	47/265 (17.7%)	
Clinicopathological variables				
Tumor grade, n/N (%)				<0.001
G1	98/530 (18.5%)	83/265 (31.3%)	15/265 (5.7%)	
G2	118/530 (22.3%)	78/265 (29.4%)	40/265 (15.1%)	
G3	314/530 (59.2%)	104/265 (39.2%)	210/265 (79.2%)	
FIGO stage, n/N (%)				0.045
Stage I	332/530 (62.6%)	181/265 (68.3%)	151/265 (57.0%)	
Stage II	49/530 (9.2%)	22/265 (8.3%)	27/265 (10.2%)	
Stage III	120/530 (22.6%)	48/265 (18.1%)	72/265 (27.2%)	
Stage IV	29/530 (5.5%)	14/265 (5.3%)	15/265 (5.7%)	
Microsatellite status, n/N (%)				0.093
MSI-H	169/530 (31.9%)	75/265 (28.3%)	94/265 (35.5%)	
MSS	361/530 (68.1%)	190/265 (71.7%)	171/265 (64.5%)	
Expression				
PLK1 expression, median [IQR]	10.24 [9.36-11.05]	9.35 [8.60-9.78]	11.05 [10.58-11.56]	<0.001

TCGA-UCEC cases were stratified into PLK1-low and PLK1-high groups according to the median PLK1 expression value. **Table 1** summarizes the 530 patient-level cases with available clinicopathological data; survival analyses separately excluded cases with follow-up <30 days and included 510 cases. Continuous variables are shown as median [IQR], and categorical variables are shown as n/N (%). *P* values were calculated using Mann-Whitney U test, chi-square test, or Fisher exact test, as appropriate. This table describes clinicopathological associations and should not be interpreted as evidence that PLK1 is an independent prognostic factor. TCGA-UCEC, The Cancer Genome Atlas uterine corpus endometrial carcinoma; PLK1, polo-like kinase 1; IQR, interquartile range; FIGO, International Federation of Gynecology and Obstetrics; MSI-H, microsatellite instability-high; MSS, microsatellite stable.

### PLK1 expression and overall survival

**Figure 2:**
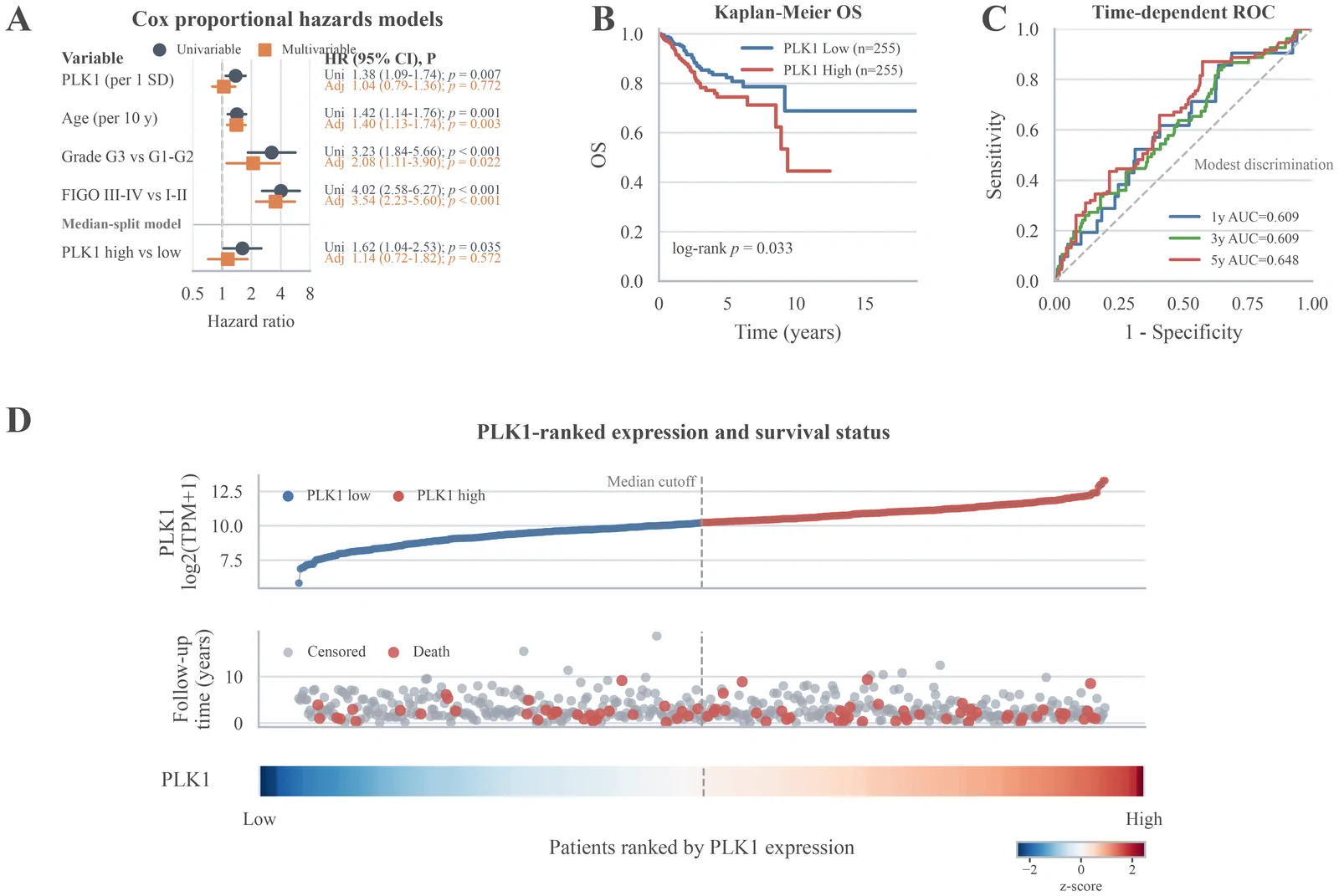
PLK1 expression is associated with overall survival in TCGA-UCEC but does not retain independent prognostic significance. **(A)** Univariable and multivariable Cox regression estimates for PLK1 modeled continuously (per 1-SD increase) and categorically (median-defined high vs. low), age (per 10-year increase), tumor grade (G3 vs. G1–G2), and FIGO stage (III–IV vs. I–II). Multivariable estimates were adjusted simultaneously for age, tumor grade, and FIGO stage. **(B)** Kaplan–Meier overall survival curves for PLK1-high and PLK1-low groups (n = 255 each; log-rank *P* = 0.033). **(C)** Time-dependent ROC curves for 1-, 3-, and 5-year overall survival (AUCs, 0.609, 0.609, and 0.648). **(D)** PLK1 expression, follow-up time, and survival status ranked by PLK1 expression. PLK1, polo-like kinase 1; TCGA, The Cancer Genome Atlas; UCEC, uterine corpus endometrial carcinoma; Uni, univariable estimate; Adj, adjusted multivariable estimate; HR, hazard ratio; CI, confidence interval; SD, standard deviation; G1–G3, histological grades 1–3; FIGO, International Federation of Gynecology and Obstetrics; OS, overall survival; ROC, receiver operating characteristic; AUC, area under the curve; TPM, transcripts per million; y, years; n, number of cases; z-score, standardized expression value.

After excluding cases with missing survival data or follow-up <30 days, 510 TCGA-UCEC cases (80 deaths) were analyzed. In univariable Cox models, PLK1 was associated with overall survival both continuously (HR per 1 SD, 1.38; 95% CI, 1.09–1.74; *P* = 0.007) and by median split (PLK1-high vs. PLK1-low: HR, 1.62; 95% CI, 1.04–2.53; *P* = 0.035). These associations were attenuated after adjustment for age, tumor grade, and FIGO stage (continuous PLK1: adjusted HR, 1.04; 95% CI, 0.79–1.36; *P* = 0.772; categorical PLK1: adjusted HR, 1.14; 95% CI, 0.72–1.82; *P* = 0.572; **Figure 2A**). No significant violation of the proportional hazards assumption was detected. Kaplan–Meier analysis using the recalculated median split (255 cases per group) showed shorter overall survival in the PLK1-high group (log-rank *P* = 0.033; **Figure 2B**). The 1-, 3-, and 5-year AUCs were 0.609, 0.609, and 0.648, respectively, indicating limited standalone discrimination (**Figure 2C**). The ranked survival-status plot is shown in **Figure 2D**.

### Functional and immune features associated with PLK1 and FOXM1

**Figure 3:**
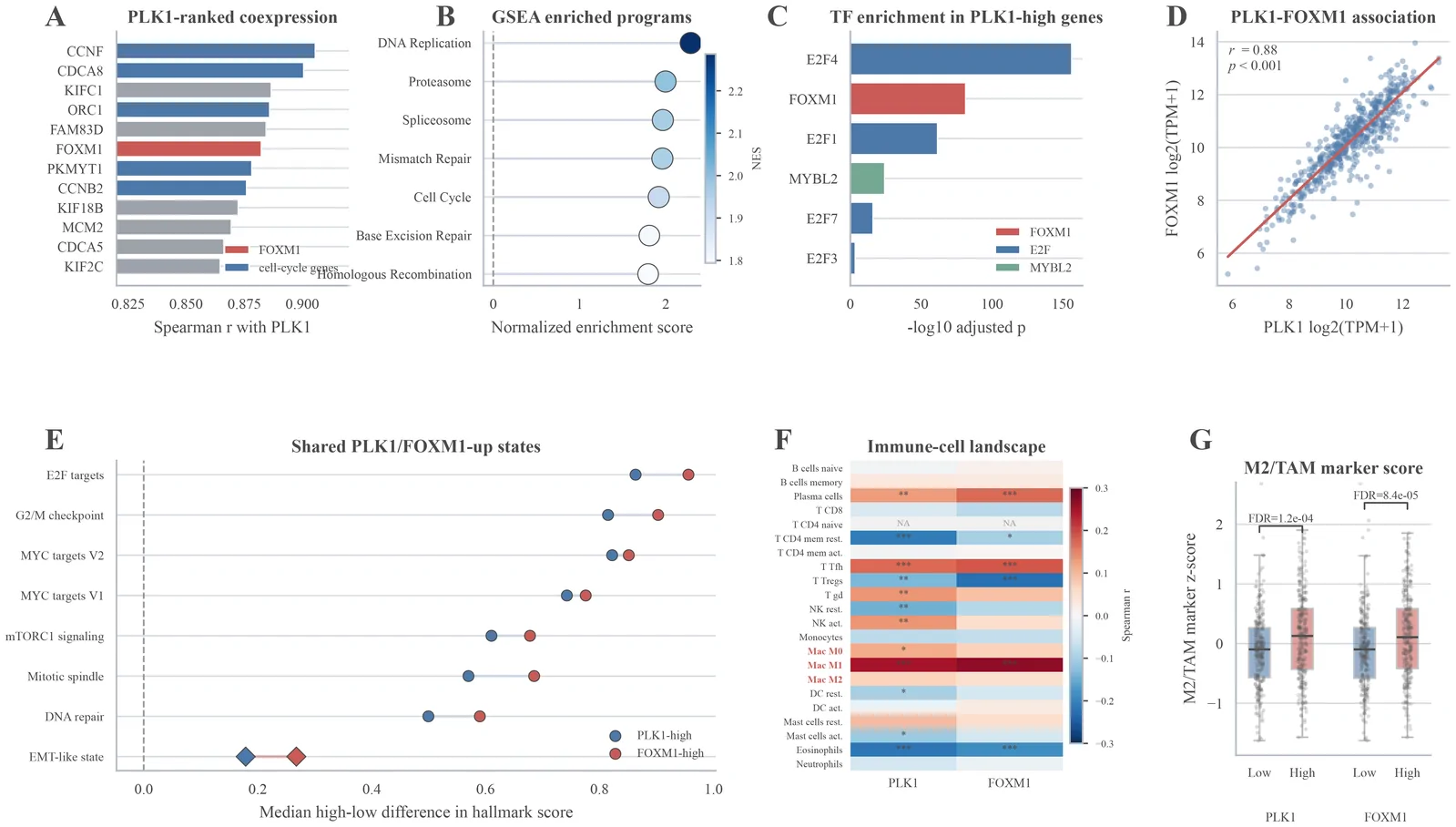
PLK1-high tumors exhibit FOXM1-associated proliferative transcriptional programs and parallel immune-contexture changes. **(A)** Whole-transcriptome genes ranked by correlation with PLK1 expression, highlighting FOXM1 and representative cell-cycle genes. **(B)** GSEA of PLK1-associated genes showing enrichment of DNA replication, cell cycle, and related programs. **(C)** Transcription-factor target enrichment of PLK1-high upregulated genes, highlighting FOXM1/E2F/MYBL2-related regulatory programs. **(D)** Correlation between PLK1 and FOXM1 expression in TCGA-UCEC. **(E)** Hallmark pathway changes commonly increased in PLK1-high and FOXM1-high tumors. **(F)** Correlation overview between PLK1/FOXM1 expression and CIBERSORT-inferred immune-cell fractions. **(G)** M2/TAM marker z-scores in PLK1-high and FOXM1-high samples. TF enrichment indicates enrichment of FOXM1/E2F target features among PLK1-high-associated genes and should not be interpreted as PLK1 acting as a transcription factor. PLK1, polo-like kinase 1; FOXM1, forkhead box M1; GSEA, gene set enrichment analysis; DNA, deoxyribonucleic acid; NES, normalized enrichment score; TF, transcription factor; E2F, E2F transcription-factor family; MYBL2, MYB proto-oncogene like 2; TCGA, The Cancer Genome Atlas; UCEC, uterine corpus endometrial carcinoma; TPM, transcripts per million; r and r_s_, Spearman rank correlation coefficients; G2/M, G2-to-mitosis transition; MYC, MYC proto-oncogene; V1 and V2, Hallmark gene-set versions 1 and 2; mTORC1, mechanistic target of rapamycin complex 1; EMT, epithelial–mesenchymal transition; CIBERSORT, Cell-type Identification By Estimating Relative Subsets Of RNA Transcripts; CD4 and CD8, clusters of differentiation 4 and 8; Tfh, T follicular helper cells; Tregs, regulatory T cells; gd, gamma-delta T cells; NK, natural killer cells; Mac, macrophages; DC, dendritic cells; mem., memory; rest., resting; act., activated; M0, unpolarized macrophages; M1 and M2, M1-like and M2-like macrophages, respectively; TAM, tumor-associated macrophage; FDR, false discovery rate; NA, not available; z-score, standardized marker score. CCNF, cyclin F; CDCA8, cell division cycle associated 8; KIFC1, kinesin family member C1; ORC1, origin recognition complex subunit 1; FAM83D, family with sequence similarity 83 member D; PKMYT1, protein kinase, membrane associated tyrosine/threonine 1; CCNB2, cyclin B2; KIF18B, kinesin family member 18B; MCM2, minichromosome maintenance complex component 2; CDCA5, cell division cycle associated 5; KIF2C, kinesin family member 2C. one, two, and three asterisks indicate FDR < 0.05, FDR < 0.01, and FDR < 0.001, respectively.

**Figure 4:**
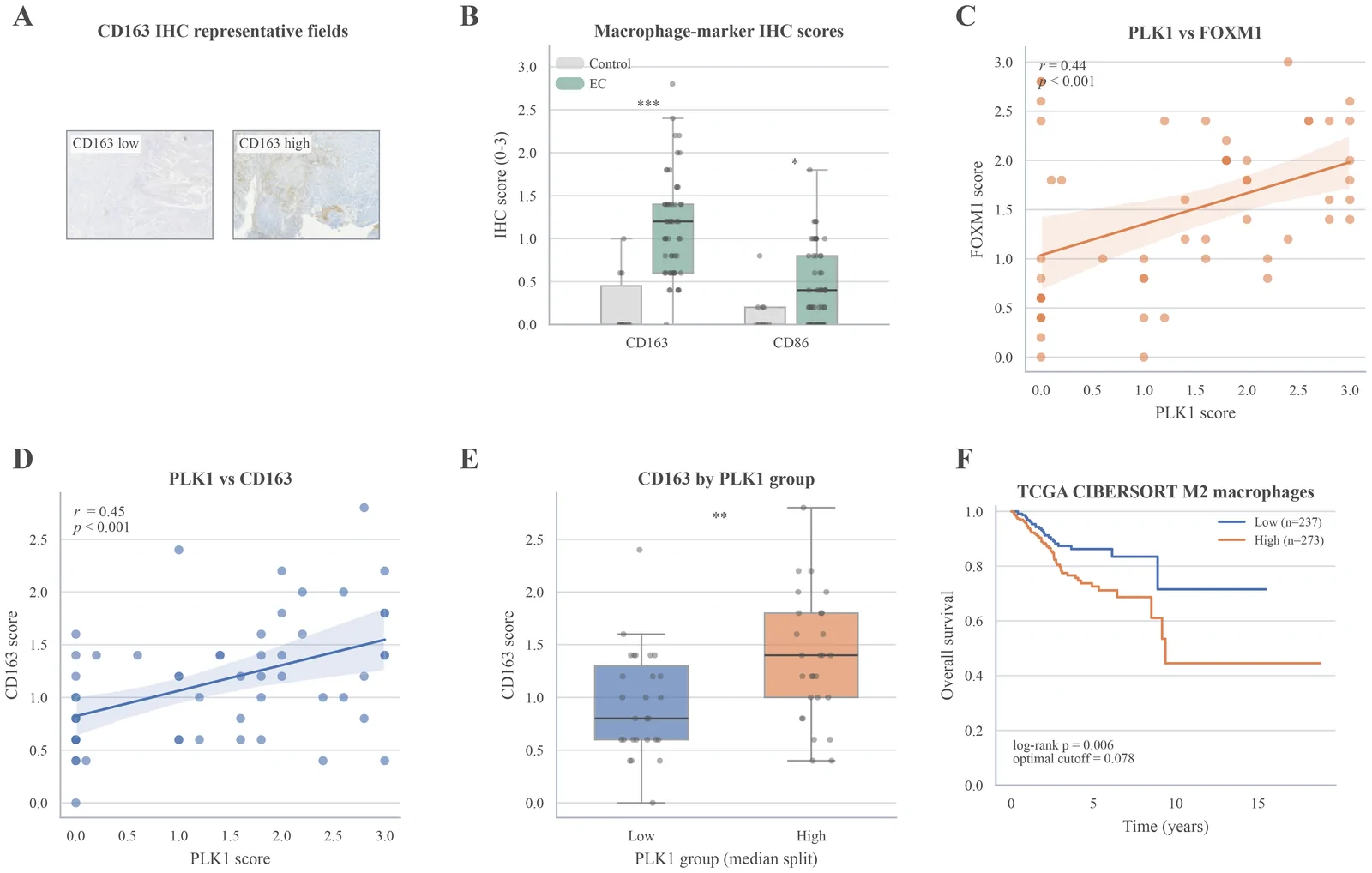
Clinical IHC validation links PLK1 expression with FOXM1 expression and CD163-positive macrophage enrichment. **(A)** Representative local IHC images showing low and high CD163-positive immune-cell infiltration. **(B)** Local IHC scores of CD163 and CD86 in EC and normal control tissues. **(C)** Correlation between PLK1 and FOXM1 IHC scores in local EC tissues. **(D)** Correlation between PLK1 and CD163 IHC scores. **(E)** CD163 IHC score comparison between PLK1 IHC-low and PLK1 IHC-high groups. **(F)** Overall survival curve for CIBERSORT-inferred Macrophages M2 high and low groups in TCGA-UCEC. Without CD68 normalization or spatial colocalization, these results are best described as CD163-positive macrophage enrichment rather than definitive M2 skewing. IHC, immunohistochemistry; PLK1, polo-like kinase 1; FOXM1, forkhead box M1; EC, endometrial cancer; CD163, cluster of differentiation 163; CD86, cluster of differentiation 86; CD68, cluster of differentiation 68; CIBERSORT, Cell-type Identification By Estimating Relative Subsets Of RNA Transcripts; M2, M2-like macrophage phenotype; TCGA, The Cancer Genome Atlas; UCEC, uterine corpus endometrial carcinoma; OS, overall survival; ρ (rho), Spearman rank correlation coefficient; n, number of cases. PLK1 IHC-low and PLK1 IHC-high groups were defined according to the median PLK1 IHC score. *P < 0.05, **P < 0.01 and ***P < 0.001.

To explore the potential functional relevance of PLK1, correlation and pathway enrichment analyses were performed using the TCGA-UCEC cohort. Whole-transcriptome correlation analysis showed that genes highly correlated with PLK1 were mainly enriched in cell-cycle and mitosis-related modules, including proliferation-associated genes such as FOXM1, CCNB1, CDK1, CDC20, BUB1B, AURKA, TOP2A, and UBE2C (**Figure 3A**). Further construction of a PLK1-centered hub module showed that this module was globally elevated in tumor tissues and was associated with higher tumor grade, advanced FIGO stage, and increased risk in univariate survival analysis (**Supplementary Figures 1A–F**). GSEA showed that PLK1-associated genes were mainly enriched in DNA replication, cell cycle, mismatch repair, homologous recombination, proteasome, and spliceosome pathways (**Figure 3B**). Transcription factor target enrichment analysis suggested enrichment of cell-cycle-related regulatory programs, including FOXM1, E2F1, E2F4, and MYBL2, among PLK1-high-associated genes, with FOXM1 emerging as a candidate transcriptional regulatory node (**Figure 3C**). In TCGA-UCEC samples, PLK1 expression was strongly positively correlated with FOXM1 expression (**Figure 3D**). Additional analyses of FOXM1 expression, clinicopathological associations, and survival are shown in **Supplementary Figure 2**. Comparison of Hallmark pathway alterations between PLK1-high and FOXM1-high tumors showed that both high-expression states were accompanied by increased E2F targets, G2/M checkpoint, MYC targets, mTORC1 signaling, mitotic spindle, and DNA repair programs. A modest increase in the EMT-like pathway score was also observed in both high-expression groups (**Figure 3E**). Immune-cell profiling showed that PLK1 and FOXM1 were correlated with multiple immune-cell infiltration estimates, including macrophage-related entries (**Figure 3F**). Z-score analysis based on the M2/TAM marker set further showed higher M2/TAM marker scores in both PLK1-high and FOXM1-high samples (**Figure 3G**). The local validation cohort included 54 EC tissues, predominantly endometrioid carcinomas (51/54, 94.4%), with most cases classified as low-grade tumors (46/54, 85.2%) and lymph node metastasis observed in 7 cases (13.0%) (**Table 2**). The histological association among PLK1, FOXM1, and macrophage markers was further evaluated in the local IHC cohort. Representative CD163 IHC images showed inter-sample differences in CD163-positive immune-cell scores (**Figure 4A**). Compared with normal controls, EC tissues showed higher CD163 and CD86 IHC scores (**Figure 4B**). PLK1 IHC scores were positively correlated with both FOXM1 and CD163 scores (**Figures 4C, D**), and PLK1-high samples exhibited higher CD163 scores than PLK1-low samples (**Figure 4E**). CD206 IHC scores also tended to be higher in PLK1 IHC-high samples. The local cohort characteristics and IHC marker scores stratified by PLK1 IHC score are summarized in **Table 2**. In addition, TCGA CIBERSORT analysis showed that the M2 macrophage-high group had shorter overall survival (**Figure 4F**). Together, these results suggest that the PLK1/FOXM1-associated tumor-cell state is primarily associated with enhanced proliferation and cell-cycle programs, accompanied by macrophage-related immune features.

**Table 2:** Local EC cohort characteristics and IHC marker scores stratified by PLK1 IHC score.

Characteristic	Overall	PLK1 IHC-low	PLK1 IHC-high	*P* value
Cases, n	54	27	27	
Normal endometrial controls, n	10			
Cases with matched clinical records, n/N	54/54			
Clinical variables				
Age, years, median [IQR]	55.00 [49.25-58.00]	55.00 [49.50-58.50]	55.00 [50.00-58.00]	0.924
BMI, median [IQR]	24.91 [23.45-28.01]	26.95 [23.14-28.82]	24.78 [23.70-25.79]	0.446
Histological subtype, n/N (%)				1.000
Endometrioid carcinoma	51/54 (94.4%)	25/27 (92.6%)	26/27 (96.3%)	
Other or unclear	3/54 (5.6%)	2/27 (7.4%)	1/27 (3.7%)	
Tumor grade, n/N (%)				0.821
High grade	6/54 (11.1%)	2/27 (7.4%)	4/27 (14.8%)	
Low grade	46/54 (85.2%)	24/27 (88.9%)	22/27 (81.5%)	
Other or unclear	2/54 (3.7%)	1/27 (3.7%)	1/27 (3.7%)	
Myometrial invasion, n/N (%)				0.221
Myometrial invasion <50%	41/53 (77.4%)	23/27 (85.2%)	18/26 (69.2%)	
No myometrial invasion	3/53 (5.7%)	2/27 (7.4%)	1/26 (3.8%)	
Myometrial invasion ≥50%	9/53 (17.0%)	2/27 (7.4%)	7/26 (26.9%)	
LVSI, n/N (%)				0.175
No	43/54 (79.6%)	24/27 (88.9%)	19/27 (70.4%)	
Yes	11/54 (20.4%)	3/27 (11.1%)	8/27 (29.6%)	
Lymph node metastasis, n/N (%)				0.100
No	47/54 (87.0%)	26/27 (96.3%)	21/27 (77.8%)	
Yes	7/54 (13.0%)	1/27 (3.7%)	6/27 (22.2%)	
Cervical invasion, n/N (%)				1.000
No	46/54 (85.2%)	23/27 (85.2%)	23/27 (85.2%)	
Yes	8/54 (14.8%)	4/27 (14.8%)	4/27 (14.8%)	
Other-site metastasis, n/N (%)				1.000
No	53/54 (98.1%)	27/27 (100.0%)	26/27 (96.3%)	
Yes	1/54 (1.9%)	0/27 (0.0%)	1/27 (3.7%)	
MMR IHC status, n/N (%)				
Any MMR protein loss	54/54 (100.0%)	27/27 (100.0%)	27/27 (100.0%)	
p53 pattern, n/N (%)				1.000
Other/abnormal pattern	1/54 (1.9%)	1/27 (3.7%)	0/27 (0.0%)	
Wild-type pattern	53/54 (98.1%)	26/27 (96.3%)	27/27 (100.0%)	
Local IHC marker scores				
PLK1 IHC score, median [IQR]	1.50 [0.00-2.20]	0.00 [0.00-1.00]	2.20 [1.80-2.80]	<0.001
FOXM1 IHC score, median [IQR]	1.50 [0.80-2.00]	0.80 [0.40-1.70]	2.00 [1.40-2.30]	<0.001
CD86 IHC score, median [IQR]	0.40 [0.00-0.80]	0.20 [0.00-0.70]	0.40 [0.00-1.00]	0.584
CD163 IHC score, median [IQR]	1.20 [0.60-1.40]	0.80 [0.60-1.30]	1.40 [1.00-1.80]	0.009
CD206 IHC score, median [IQR]	0.20 [0.00-0.60]	0.00 [0.00-0.20]	0.40 [0.20-0.80]	0.005

The local EC cohort was stratified into PLK1 IHC-low and PLK1 IHC-high groups according to the median PLK1 IHC score among 54 cancer tissues. Ten normal endometrial controls were obtained from cervical cancer patients and were used for tissue expression validation, but were not included in PLK1-high/low clinical comparisons. Continuous variables are shown as median [IQR], and categorical variables are shown as n/N (%). IHC scores were assigned using a 0–3 marker-positive cell-density scale based on the average number of positive cells across five 400× high-power fields. BMI, body mass index; EC, endometrial cancer; IHC, immunohistochemistry; IQR, interquartile range; LVSI, lymphovascular space invasion; MMR, mismatch repair; PLK1, polo-like kinase 1.

### PLK1 and FOXM1 interaction and Thr600 phosphorylation

**Figure 5:**
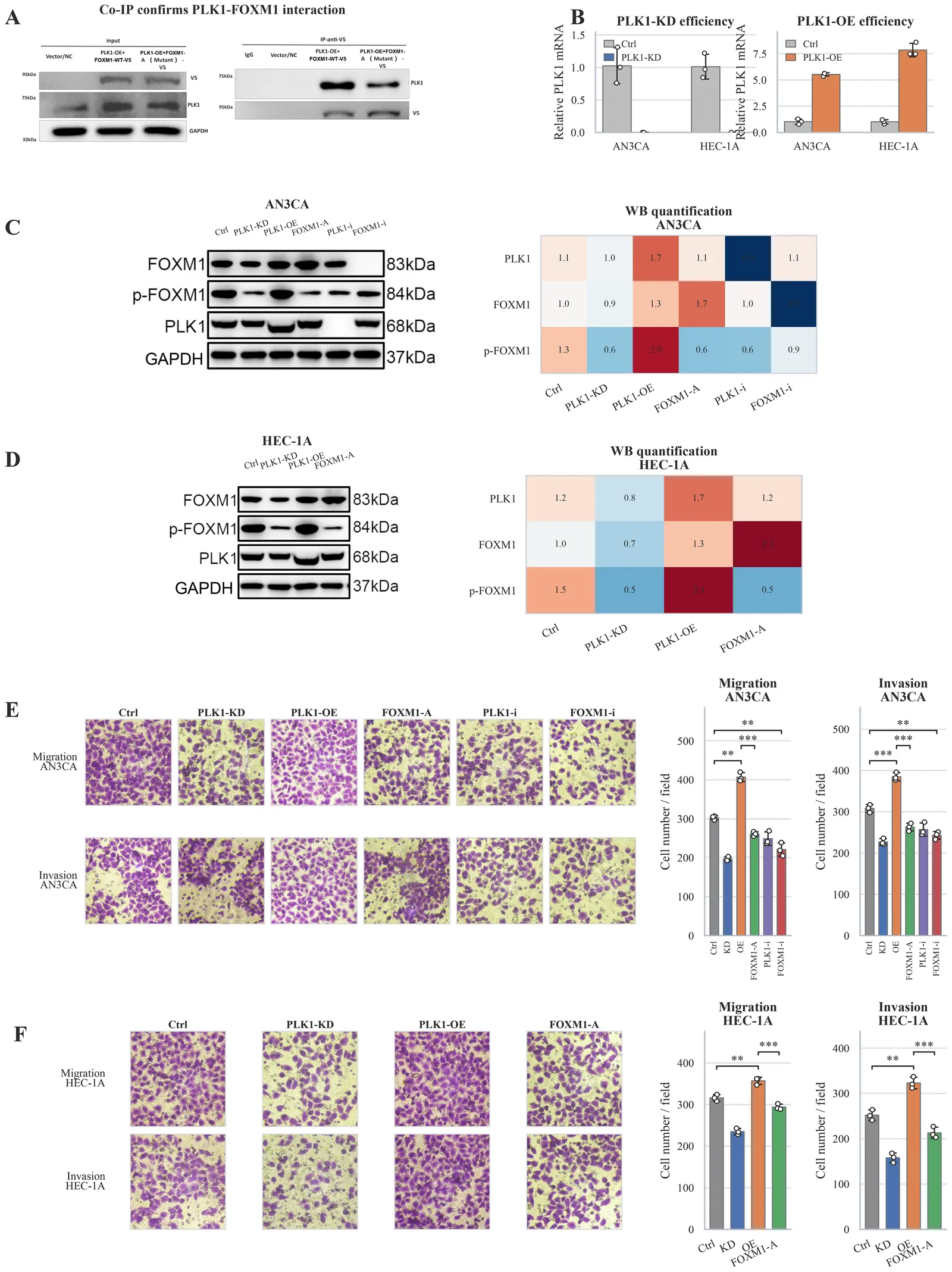
FOXM1 Thr600 phosphorylation-related alterations in EC cell migration and invasion phenotypes. **(A)** Co-IP assay supporting PLK1–FOXM1 interaction. **(B)** qRT-PCR validation of PLK1 knockdown and overexpression efficiency in AN3CA and HEC-1A cells. **(C)** Representative Western blot image and densitometric summary of PLK1, FOXM1, and p-FOXM1(Thr600) in AN3CA cells. **(D)** Representative Western blot image and densitometric summary of PLK1, FOXM1, and p-FOXM1(Thr600) in HEC-1A cells. **(E)** Representative Transwell migration and invasion images and quantification in AN3CA cells. **(F)** Representative Transwell migration and invasion images and quantification in HEC-1A cells. PLK1, polo-like kinase 1; FOXM1, forkhead box M1; p-FOXM1(Thr600), FOXM1 phosphorylated at threonine 600; EC, endometrial cancer; Co-IP, co-immunoprecipitation; IP, immunoprecipitation; IgG, immunoglobulin G; NC, negative control; V5, V5 epitope tag; WT, wild type; AN3CA and HEC-1A, human endometrial cancer cell lines; qRT-PCR, quantitative reverse-transcription polymerase chain reaction; mRNA, messenger RNA; Ctrl, control; KD, knockdown; OE, overexpression; FOXM1-A, experimental label for the FOXM1-T600A mutant; T600A, threonine-to-alanine substitution at residue 600; PLK1-i, BI 2536 treatment; FOXM1-i, FDI-6 treatment; WB, Western blotting; GAPDH, glyceraldehyde-3-phosphate dehydrogenase; kDa, kilodalton; SD, standard deviation. PLK1 and total FOXM1 band intensities were normalized to GAPDH, whereas p-FOXM1(Thr600) was normalized to total FOXM1. Data are presented as mean ± SD from three independent experiments. **P < 0.01 and ***P < 0.001.

Co-IP analysis showed that, under PLK1-OE conditions, PLK1 signals were detected in the immunoprecipitates of both FOXM1-WT-V5 and FOXM1-T600A-V5, whereas no obvious PLK1 co-precipitation was observed in the IgG control group. These findings suggest that PLK1 interacts with FOXM1 and that the T600A mutation does not substantially disrupt PLK1–FOXM1 interaction (**Figure 5A**). This experiment was used to verify the presence of PLK1–FOXM1 interaction and was not interpreted as a comparison of binding differences between FOXM1-WT and FOXM1-T600A. qRT-PCR analysis confirmed that PLK1-KD decreased PLK1 mRNA expression, whereas PLK1-OE increased PLK1 mRNA expression in both AN3CA and HEC-1A cells (**Figure 5B**). Western blotting showed that PLK1-OE increased p-FOXM1(Thr600) levels, whereas PLK1-KD decreased p-FOXM1(Thr600) levels in AN3CA and HEC-1A cells. In the FOXM1-T600A group, total FOXM1 remained detectable, but p-FOXM1(Thr600) levels were lower than those in the PLK1-OE group, consistent with the Thr600 phospho-deficient design (**Figures 5C, D**). In AN3CA cells, PLK1-i treatment reduced p-FOXM1(Thr600) levels, whereas FOXM1-i treatment decreased total FOXM1 expression (**Figure 5C**). Transwell assays further showed that, in AN3CA cells, PLK1-OE increased the numbers of migrated and invaded cells, whereas PLK1-KD, FOXM1-T600A, PLK1-i, and FOXM1-i reduced these phenotypes (**Figure 5E**). In HEC-1A cells, PLK1-OE similarly enhanced migration and invasion, whereas PLK1-KD and FOXM1-T600A weakened these phenotypes (**Figure 5F**). These results support an association between FOXM1 Thr600 phosphorylation-related alterations and migration and invasion phenotypes in EC cells.

### Association between the PLK1/FOXM1-associated tumor-cell state and macrophage-like phenotype changes

**Figure 6:**
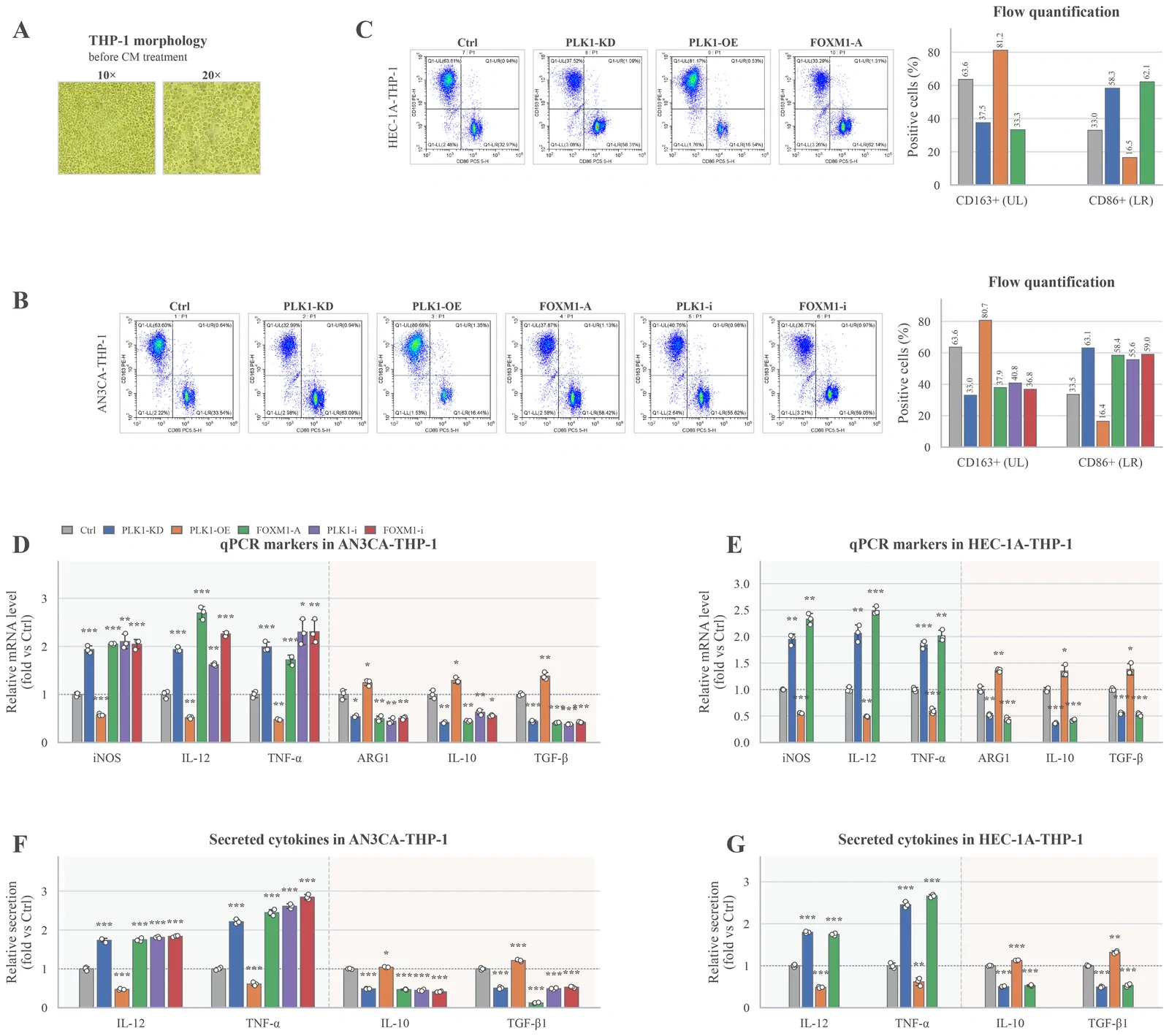
Association of the PLK1/FOXM1-associated tumor-cell state with M2-like marker changes in THP-1-derived macrophage-like cells. **(A)** Representative morphology of PMA-induced THP-1-derived macrophage-like cells before conditioned-medium (CM) treatment. **(B)** Representative CD86/CD163 flow cytometry plots and quantification in the AN3CA–THP-1 conditioned-medium system. **(C)** Representative CD86/CD163 flow cytometry plots and quantification in the HEC-1A–THP-1 conditioned-medium system. **(D, E)** qRT-PCR analysis of M1-like and M2-like macrophage marker genes in AN3CA–THP-1 and HEC-1A–THP-1 systems. **(F, G)** ELISA analysis of secreted cytokines in AN3CA–THP-1 and HEC-1A–THP-1 systems. PLK1-OE tumor-cell conditions were associated with increased expression of M2-like markers, whereas PLK1-KD, FOXM1-T600A, PLK1-i, or FOXM1-i attenuated this phenotype. PLK1, polo-like kinase 1; FOXM1, forkhead box M1; THP-1, human monocytic leukemia cell line; AN3CA and HEC-1A, human endometrial cancer cell lines; PMA, phorbol 12-myristate 13-acetate; CM, conditioned medium; CD86, cluster of differentiation 86; CD163, cluster of differentiation 163; PE, phycoerythrin; PC5, phycoerythrin-cyanine 5; H, fluorescence-signal height; P1, analyzed parent-population gate; Q1, quadrant-gate identifier; UL, upper-left quadrant; UR, upper-right quadrant; LL, lower-left quadrant; LR, lower-right quadrant; +, marker-positive cells; Ctrl, control; KD, knockdown; OE, overexpression; FOXM1-A, experimental label for the FOXM1-T600A mutant; T600A, threonine-to-alanine substitution at residue 600; PLK1-i, BI 2536 treatment; FOXM1-i, FDI-6 treatment; qPCR, quantitative polymerase chain reaction; qRT-PCR, quantitative reverse-transcription polymerase chain reaction; mRNA, messenger RNA; M1 and M2, M1-like and M2-like macrophage phenotypes, respectively; iNOS, inducible nitric oxide synthase; IL, interleukin; TNF-α, tumor necrosis factor alpha; ARG1, arginase 1; TGF-β, transforming growth factor beta; ELISA, enzyme-linked immunosorbent assay; SD, standard deviation. The labels 10× and 20× indicate objective magnification. Flow-cytometry percentages correspond to the representative plots shown. qRT-PCR and ELISA data are presented as mean ± SD from three independent experiments. *P < 0.05, **P < 0.01, and ***P < 0.001 versus Ctrl.

To assess the relationship between the PLK1/FOXM1-associated tumor-cell state and macrophage polarization phenotypes, a conditioned-medium-induced THP-1 macrophage-like cell model was used. The morphology of PMA-induced THP-1 cells is shown in **Figure 6A**. Flow cytometry showed that, in the AN3CA–THP-1 system, conditioned medium from PLK1-OE cells increased the proportion of CD163-positive cells and decreased the proportion of CD86-positive cells. In contrast, the PLK1-KD, FOXM1-T600A, PLK1-i, and FOXM1-i groups showed opposite or attenuated trends (**Figure 6B**). Similar changes were observed in the HEC-1A–THP-1 system, in which the PLK1-OE group showed an increased CD163-positive proportion and a decreased CD86-positive proportion, whereas the PLK1-KD and FOXM1-T600A groups showed the opposite pattern (**Figure 6C**). Polarization-related gene expression analysis showed that, in the AN3CA–THP-1 system, PLK1-OE decreased the expression of M1-like markers, including NOS2, IL12A, and TNF, and increased the expression of M2-like markers, including ARG1, IL10, and TGFB1. PLK1-KD, FOXM1-T600A, and inhibitor-treated groups showed overall changes in the opposite direction (**Figure 6D**). In the HEC-1A–THP-1 system, PLK1-OE and PLK1-KD/FOXM1-T600A groups also showed opposite expression patterns of M1-like and M2-like markers (**Figure 6E**). ELISA further showed that, in both AN3CA–THP-1 and HEC-1A–THP-1 systems, PLK1-OE conditions reduced IL-12p70 and TNF-α secretion, whereas IL-10 and TGF-β1 secretion was increased. PLK1-KD, FOXM1-T600A, and inhibitor-treated groups generally showed opposite trends (**Figures 6F, G**). These findings suggest that conditioned medium from tumor cells exhibiting the PLK1/FOXM1-associated tumor-cell state was associated with altered M2-like marker profiles in THP-1-derived macrophage-like cells. This model was used to evaluate macrophage polarization phenotypes and does not demonstrate that polarized macrophages subsequently feedback to regulate tumor-cell migration or invasion.

### Computational therapeutic prioritization for PLK1-high tumors

**Figure 7:**
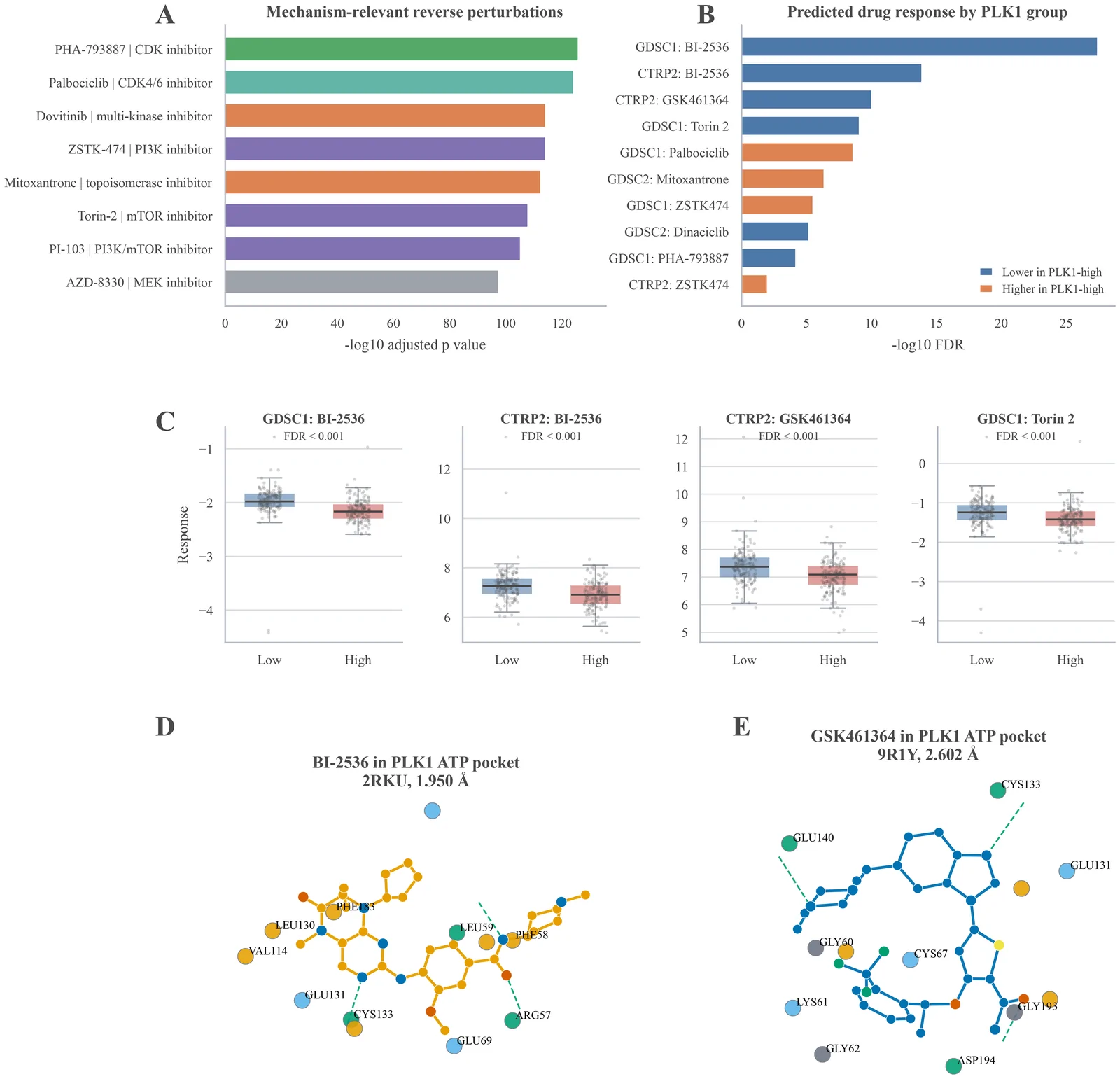
Computational therapeutic prioritization of PLK1-high EC. **(A)** Mechanism-relevant inverse perturbation results for the PLK1-high transcriptional signature. **(B)** oncoPredict-based drug-response differences between PLK1-high and PLK1-low tumors. **(C)** Representative predicted response distributions for selected PLK1-targeting or cell-cycle-related candidate drugs. **(D)** Structure-supported view of BI-2536 in the PLK1 ATP-binding pocket based on 2RKU. **(E)** Structure-supported view of GSK461364 in the PLK1 ATP-binding pocket based on 9R1Y. PLK1, polo-like kinase 1; EC, endometrial cancer; CDK, cyclin-dependent kinase; CDK4/6, cyclin-dependent kinases 4 and 6; PI3K, phosphoinositide 3-kinase; mTOR, mechanistic target of rapamycin; MEK, mitogen-activated protein kinase kinase; GDSC1 and GDSC2, Genomics of Drug Sensitivity in Cancer datasets 1 and 2, respectively; CTRP2, Cancer Therapeutics Response Portal version 2; FDR, false discovery rate; ATP, adenosine triphosphate; PDB, Protein Data Bank; Å, angstrom; −log10, negative base-10 logarithm. Amino-acid residue abbreviations are as follows: CYS, cysteine; GLU, glutamic acid; PHE, phenylalanine; LEU, leucine; VAL, valine; ARG, arginine; GLY, glycine; LYS, lysine; ASP, aspartic acid. PLK1-high and PLK1-low groups were defined according to the median PLK1 expression level. Within each training dataset, lower predicted response values indicate greater predicted sensitivity; absolute response values should not be compared directly across datasets. These computational analyses are hypothesis-generating and do not constitute experimental validation of drug efficacy.

Inverse perturbation analysis based on the PLK1-high transcriptional signature identified multiple classes of candidate small molecules, mainly involving cyclin-dependent kinase (CDK) inhibitors, CDK4/6 inhibitors, phosphoinositide 3-kinase/mechanistic target of rapamycin (PI3K/mTOR) inhibitors, mTOR inhibitors, and PLK1-related inhibitors (**Figure 7A**). oncoPredict-based drug response analysis showed that PLK1-high and PLK1-low samples differed in predicted responses to several candidate drugs. In the response scoring system shown in **Figures 7B, C**, lower predicted response values indicate higher predicted sensitivity; BI-2536, GSK461364, Torin 2, Dinaciclib, and PHA-793887 generally showed lower predicted response values in the PLK1-high group (**Figures 7B, C**). Structure-supported analysis further showed that BI-2536 and GSK461364 were located in PLK1 adenosine triphosphate (ATP)-binding pocket-related complex structures 2RKU and 9R1Y, respectively, and formed potential hydrogen bonds and hydrophobic interactions with surrounding pocket residues (**Figures 7D, E**). These findings nominate PLK1 inhibitors and cell-cycle-related inhibitors as candidate therapeutic directions for PLK1-high EC, although this part of the analysis remains computational and requires further experimental validation.

## Discussion

Using public datasets, a local IHC cohort, and *in vitro* functional assays, this study evaluated PLK1 in EC at the clinicopathological, transcriptomic, and cellular functional levels. PLK1 was consistently upregulated and associated with higher tumor grade and FIGO stage. Functional experiments supported an interaction between PLK1 and FOXM1, and suggested that FOXM1 Thr600 phosphorylation-related alterations were associated with altered migration and invasion phenotypes. In addition, the PLK1/FOXM1-associated tumor-cell state was linked to macrophage-related immune features and was associated with changes in the M2-like marker profile of THP-1-derived cells.

PLK1 has been implicated in aggressive behavior across several solid tumors, including intrahepatic cholangiocarcinoma and epithelial ovarian cancer, where PLK1-related programs have been linked to invasion, epithelial adhesion disruption, proliferative activity, DNA damage, apoptosis, and tumor growth (32, 33). Consistently, our findings show that PLK1 is upregulated in EC and correlates with tumor grade and stage. PLK1 inhibitors such as BI-2536 and GSK461364 provide pharmacological support for PLK1 targeting in mitotic cancer models (34, 35). In addition, CDK4/6 inhibition and mTOR pathway inhibition have EC-specific therapeutic evidence (36, 37). However, most previous studies have focused on single-target pharmacological effects, with limited integration of PLK1-associated transcriptional states and tumor microenvironment features. In contrast, this study characterizes a PLK1/FOXM1-associated tumor-cell state linked to proliferative and invasion-related programs. FOXM1 has been linked to oncogenic transcriptional programs and migration and invasion phenotypes (38, 39). Our findings further suggest that FOXM1 represents a key molecular component of this PLK1/FOXM1-associated tumor-cell state and may contribute to the connection between proliferative and invasion-related phenotypes. However, the biological functions of PLK1 and FOXM1 may vary depending on tumor context. Although both proteins are frequently associated with proliferative and oncogenic programs, context-dependent functions have also been described, including tumor-suppressive properties reported in parts of the FOXM1 literature; their functional consequences may be influenced by tumor lineage, genetic background, and the surrounding regulatory environment (7, 14, 38). Therefore, the PLK1/FOXM1-associated tumor-cell state identified in this study should be interpreted within the specific molecular context of EC rather than as a universal mechanism across epithelial malignancies. The PLK1/FOXM1-associated tumor-cell state in EC is characterized by coordinated activation of cell-cycle and E2F-associated transcriptional programs. This state is characterized by sustained expression of PLK1 and mitotic regulators such as CCNB1 and CDK1, supporting persistent proliferative signaling. Within this context, FOXM1 may represent a transcriptional component associated with both proliferative and invasive phenotypes. FOXM1-related molecular features within this state may accompany invasion-related transcriptional programs and are associated with tumor cell migration and invasion phenotypes. In parallel, this tumor-cell state may be associated with changes in tumor-immune interactions. Tumor-derived metabolic and inflammatory cues can shape macrophage polarization and tumor-promoting immune contexture (40, 41). Our findings suggest that the PLK1/FOXM1-associated tumor-cell state may be associated with such immune contexture changes, although a direct causal relationship remains to be established.

This study integrates multi-layered datasets, including transcriptomic, proteomic, histopathological, and *in vitro* functional evidence, to characterize PLK1 in EC. The integration of PLK1, FOXM1, cell-cycle programs, and immune-related features enables a more comprehensive interpretation of the PLK1/FOXM1-associated tumor-cell state. Additionally, experimental validation provides functional support beyond purely computational analyses.

Several limitations should be acknowledged. First, public datasets are retrospective and may be affected by batch effects and sample heterogeneity. Second, the local IHC cohort is relatively small and relies on semi-quantitative scoring without spatial or CD68-based normalization. Third, the THP-1 conditioned-medium model used in this study represents an exploratory *in vitro* system to evaluate tumor-cell-associated changes in macrophage-like phenotypes but cannot fully recapitulate the heterogeneity and complexity of primary tumor-associated macrophages within the tumor microenvironment. Moreover, macrophage-related changes were evaluated using a limited panel of polarization-associated markers, which may not capture the full spectrum of macrophage states. Future studies using primary macrophages, patient-derived models, and single-cell or spatial transcriptomic approaches are needed to further validate the relationship between the PLK1/FOXM1-associated tumor-cell state and macrophage remodeling. Fourth, although the FOXM1-T600A construct supports a Thr600 phosphorylation-related phenotype, the upstream kinase relationship between PLK1 and FOXM1 Thr600 phosphorylation still requires direct biochemical validation. Furthermore, this study supports a biologically plausible association among the PLK1/FOXM1-associated tumor-cell state, migration and invasion phenotypes, and macrophage-related immune features, but does not establish a complete causal signaling hierarchy *in vivo*. Finally, drug response prediction analyses were based on computational modeling and structure-supported prioritization rather than direct pharmacological experiments. Given the potential for false-positive predictions in perturbation-based screening, the identified compounds should be regarded as hypothesis-generating candidates and require further validation using cellular drug-response assays, *in vivo* models, and, ultimately, clinical studies. Future studies should include *in vivo* models, primary macrophage systems, and spatial transcriptomics to further validate the relationship between the PLK1/FOXM1-associated tumor-cell state and tumor–immune interactions. Proteomic and biochemical assays are also required to confirm FOXM1 phosphorylation mechanisms and evaluate therapeutic responses to PLK1-targeted agents.

## Conclusion

PLK1 is significantly upregulated in EC and is associated with cell-cycle activation and invasive phenotypes. The PLK1/FOXM1-associated tumor-cell state may represent a distinct molecular feature linked to immune microenvironment alterations, providing a framework for further mechanistic studies and evaluation of potential therapeutic strategies.

## Data Availability

The original contributions presented in the study are included in the article/**Supplementary Material**. Further inquiries can be directed to the corresponding author.
