# Supplementary material for: PLK1/FOXM1-associated tumor-cell state and macrophage-related immune features in endometrial cancer
Source: Front Oncol. 2026 Aug 26;16:1931435. doi: 10.3389/fonc.2026.1931435 (PMC13553901; doi:10.3389/fonc.2026.1931435)
Supplement: Supplementary file 1 [file DataSheet1.pdf]

Supplementary Materials

Supplementary figures and tables for: PLK1/FOXM1-associated tumor-cell state and macrophage-related immune features in endometrial cancer.

Supplementary Figures

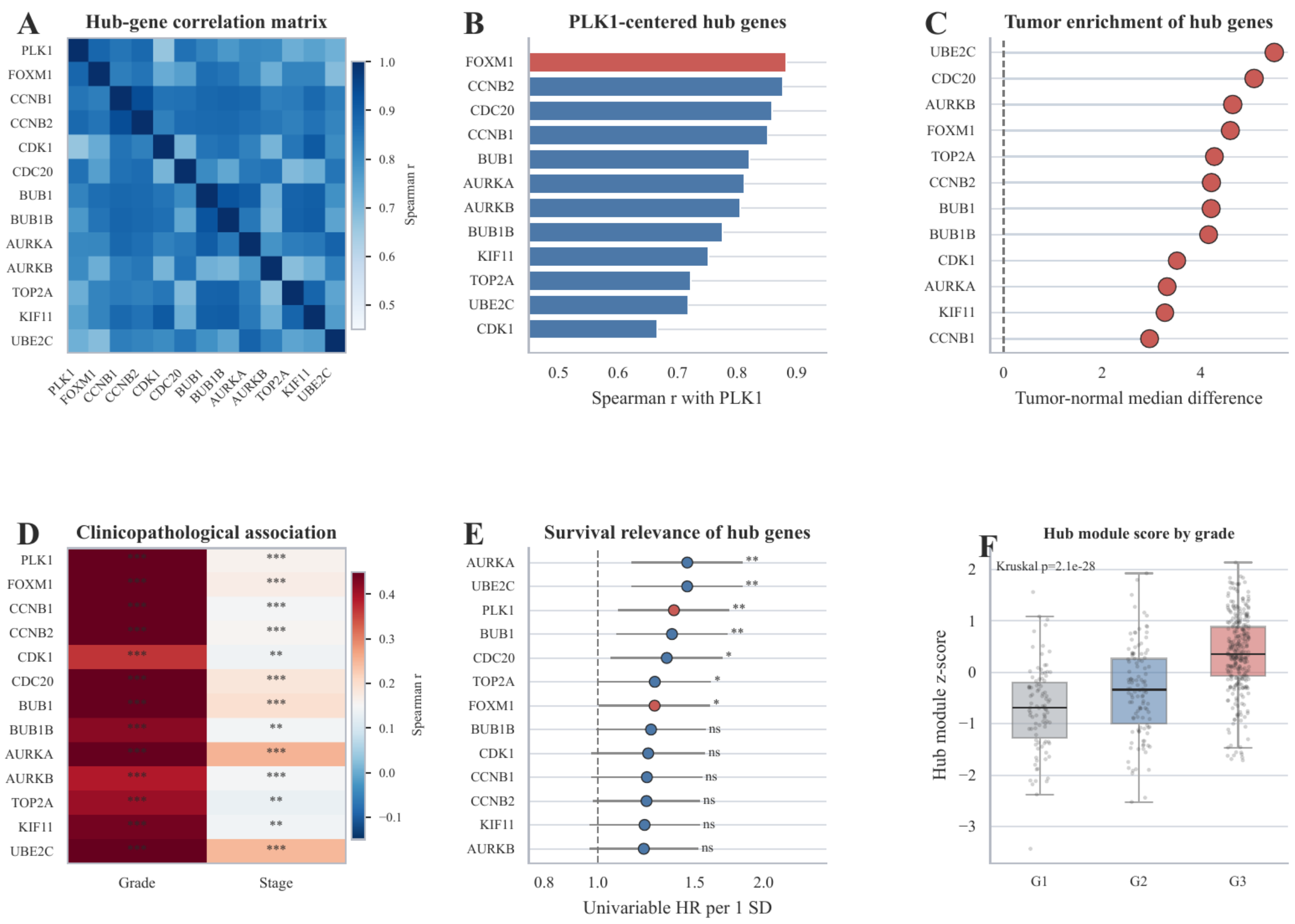

Supplementary Figure 1. PLK1-associated hub module confirms proliferation-related coexpression and clinical confounding. (A) Correlation matrix of PLK1 and cell-cycle hub genes. (B) PLK1-centered Spearman correlations with representative hub genes. (C) Tumor-normal expression differences of hub genes. (D) Associations between the hub module and clinicopathological features. (E) Univariate Cox survival associations of hub genes. (F) Hub-module z-score distributions across clinical strata.

Note: PLK1, polo-like kinase 1; FOXM1, forkhead box M1; CCNB1 and CCNB2, cyclins B1 and B2; CDK1, cyclin-dependent kinase 1; CDC20, cell division cycle 20; BUB1, BUB1 mitotic checkpoint serine/threonine kinase; BUB1B, BUB1 mitotic checkpoint serine/threonine kinase B; AURKA and AURKB, aurora kinases A and B; TOP2A, DNA topoisomerase II alpha; KIF11, kinesin family member 11; UBE2C, ubiquitin-conjugating enzyme E2 C; r, Spearman rank correlation coefficient; HR, hazard ratio; CI, confidence interval; SD, standard deviation; G1–G3, histological grades 1–3; z-score, standardized expression score; ns, not significant. The hub-module score was calculated as the mean z-standardized expression of the displayed module genes, excluding PLK1. In panel E, points represent HR estimates and horizontal lines represent 95% CIs. Statistical significance is indicated as $*P < 0.05$, $**P < 0.01$ and $***P < 0.001$. The overall difference in panel F was evaluated using the Kruskal–Wallis test.

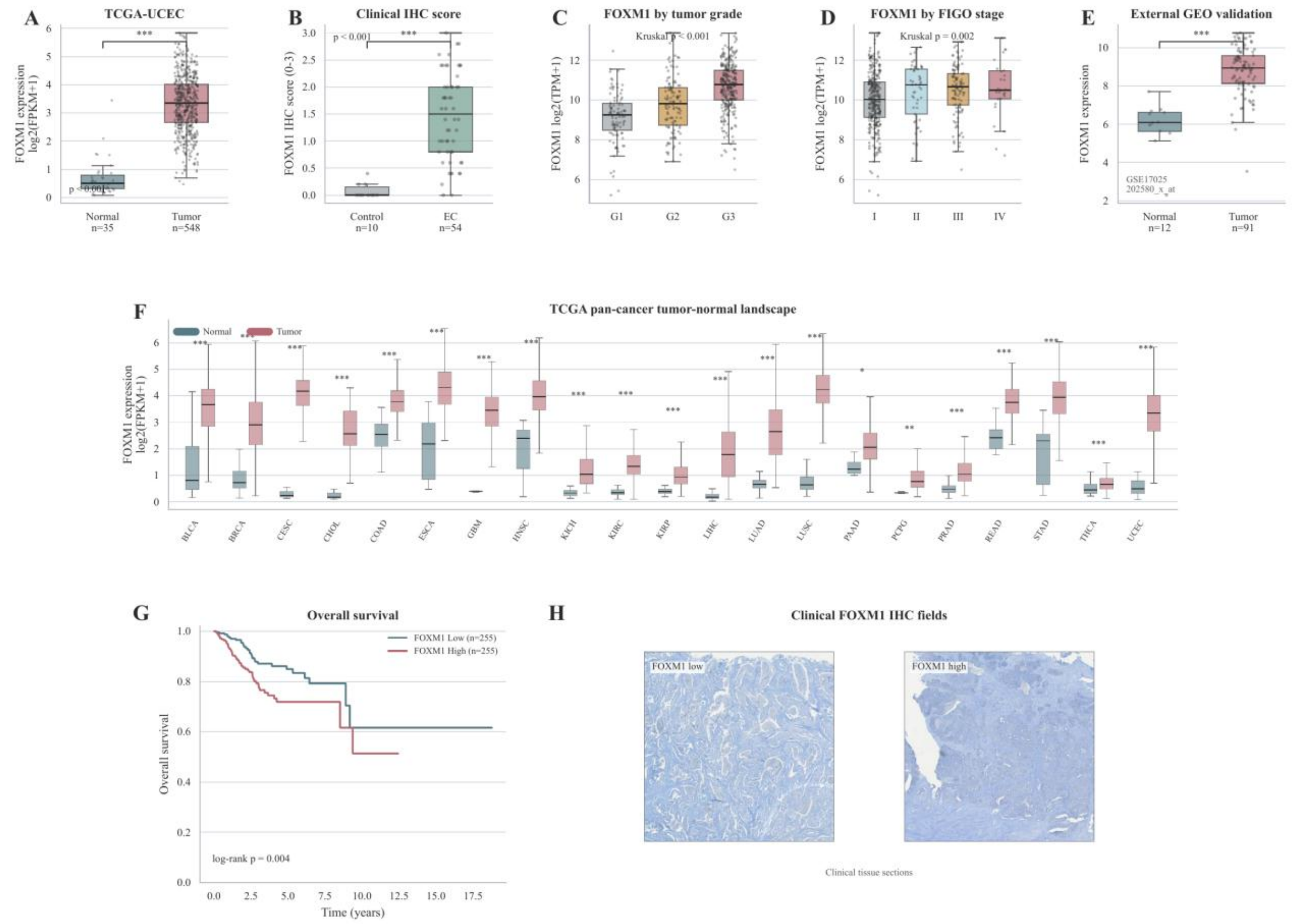

Supplementary Figure 2. FOXM1 public-database and clinical IHC evidence in endometrial cancer. (A) FOXM1 expression in TCGA-UCEC tumor and normal tissues. (B) Local FOXM1 IHC score comparison. (C, D) Association of FOXM1 expression with tumor grade and FIGO stage. (E) External validation of FOXM1 expression in GSE17025. (F) Pan-cancer tumor-normal expression profile of FOXM1. (G) Overall survival curve stratified by FOXM1 expression in TCGA-UCEC. (H) Representative local FOXM1-low and FOXM1-high IHC images. These data support FOXM1 as a candidate PLK1-high-associated molecular feature rather than an independent prognostic marker.

Note: FOXM1, forkhead box M1; TCGA, The Cancer Genome Atlas; UCEC, uterine corpus endometrial carcinoma; FPKM, fragments per kilobase of transcript per million mapped reads; TPM, transcripts per million; IHC, immunohistochemistry; EC, endometrial cancer; FIGO, International Federation of Gynecology and Obstetrics; G1–G3, histological grades 1–3; GEO, Gene Expression Omnibus; GSE17025, GEO Series accession GSE17025; 202580_x_at, Affymetrix probe-set identifier for FOXM1; OS, overall survival; n, number of samples or cases. TCGA cancer-type abbreviations are as follows: BLCA, bladder urothelial carcinoma; BRCA, breast invasive carcinoma; CESC, cervical squamous cell carcinoma and endocervical adenocarcinoma; CHOL, cholangiocarcinoma; COAD, colon adenocarcinoma; ESCA, esophageal carcinoma; GBM, glioblastoma multiforme; HNSC, head and neck squamous cell carcinoma; KICH, kidney chromophobe; KIRC, kidney renal clear cell carcinoma; KIRP, kidney renal papillary cell carcinoma; LIHC, liver hepatocellular carcinoma; LUAD, lung adenocarcinoma; LUSC, lung squamous cell carcinoma; PAAD, pancreatic adenocarcinoma; PCPG, pheochromocytoma and paraganglioma; PRAD, prostate adenocarcinoma; READ, rectum adenocarcinoma; STAD, stomach adenocarcinoma; THCA, thyroid carcinoma. FOXM1-high and FOXM1-low groups were defined according to the median FOXM1 expression level. Statistical significance is indicated as *$P < 0.05$, **$P < 0.01$ and ***$P < 0.001$.

Supplementary Tables

Supplementary Table 1. qPCR primer sequences and construct/plasmid information status

Item type	Target or construct	Forward sequence (5'-3')	Reverse sequence (5'-3')	Notes/status
qPCR primer	PLK1	CGTCCCCGGGAAAAAGAAGA	CAGGATCCTCAGCCTCCTCT	Used for PLK1 knockdown/overexpression efficiency validation
qPCR primer	iNOS	CGCATGACCTTGGTGTTTGG	CATAGACCTTGGGCTTGCCA	Macrophage polarization marker
qPCR primer	IL-12	GCACAGTGGAGGCCTGTTTA	GCCAGGCAACTCCCATTAGT	Macrophage polarization marker
qPCR primer	TNF-α	CTTCTCGAACCCCGAGTGAC	ATGAGGTACAGGCCCTCTGA	Macrophage polarization marker
qPCR primer	ARG1	GTCTGTGGGAAAAGCAAGCG	CACCAGGCTGATTCTTCCGT	Macrophage polarization marker
qPCR primer	IL-10	CGAGATGCCTTCAGCAGAGT	CGCCTTGATGTCTGGGTCTT	Macrophage polarization marker
qPCR primer	TGF-β	GTGGACATCAACGGGTTCAC	ATGAGAAGCAGGAAAGGCCG	Macrophage polarization marker
qPCR primer	GAPDH	GGAGAGTGTTTCCTCGTCCC	ATGAAGGGGTCGTTGATGGC	Internal control; same sequence also appears in the PLK1 efficiency qPCR report
Construct/plasmid information	PLK1-KD			Group name appears in experimental files; vector backbone, supplier/catalog, and shRNA/siRNA target sequence were not found in current source files
Construct/plasmid information	PLK1-OE			Group name appears in experimental files; vector backbone, supplier/catalog, and PLK1 insert/accession details were not found in current source files
Construct/plasmid information	FOXM1-WT-V5/3*FLAG	NA	NA	pCDNA3.1-CMV-V5-FOXM1 (human) wild-type-3*FLAG vector record (E-1146; source record: E-1146 wild-type vector record).
Construct/plasmid information	FOXM1-T600A-V5/3*FLAG	NA	NA	pCDNA3.1-CMV-V5-FOXM1 (human) T600A-3*FLAG vector record (E-1147; source record: E-1147 T600A vector record); threonine 600 was substituted with alanine.

Note: This table summarizes qPCR primer sequences used for PLK1 transfection-efficiency validation and THP-1 macrophage-polarization marker assays, together with FOXM1-WT and FOXM1-T600A plasmid information. qPCR, quantitative polymerase chain reaction; PLK1, polo-like kinase 1; THP-1, human monocytic leukemia cell line; iNOS, inducible nitric oxide synthase; IL, interleukin; TNF-α, tumor necrosis factor alpha; ARG1, arginase 1; TGF-β, transforming growth factor beta; GAPDH, glyceraldehyde-3-phosphate dehydrogenase; KD, knockdown; OE, overexpression; FOXM1, forkhead box M1; WT, wild type; T600A, threonine-to-alanine substitution at FOXM1 residue 600; V5, V5 epitope tag; 3×FLAG, triple FLAG epitope tag; CMV, cytomegalovirus promoter; shRNA, short hairpin RNA; siRNA, small interfering RNA; NA, not applicable. Primer sequences are presented in the 5′-to-3′ direction. Blank cells indicate information that was unavailable in the source records.

Supplementary Table 2. Primary antibodies and IHC detection reagents

Category	Target/Reagent	Host/type	Clone	Catalog no.	Supplier	Application	Notes
Primary antibody	anti-PLK1 rabbit mAb	Rabbit monoclonal antibody	208G4	4513S/4513T	Shanghai Universal Biotech Co., Ltd.	IHC	Used for PLK1 immunohistochemistry in local tissue sections
Primary antibody	anti-FOXM1 rabbit mAb	Rabbit monoclonal antibody	D3F2B	20459T	Shanghai Universal Biotech Co., Ltd.	IHC	Used for FOXM1 immunohistochemistry in local tissue sections
Primary antibody	anti-CD86 rabbit mAb	Rabbit monoclonal antibody	E2G8P	91882S	Shanghai Universal Biotech Co., Ltd.	IHC	Used for CD86 immunohistochemistry in local tissue sections
Primary antibody	anti-CD163 rabbit mAb	Rabbit monoclonal antibody	D6U1J	93498T	Shanghai Universal Biotech Co., Ltd.	IHC	Used for CD163 immunohistochemistry in local tissue sections
Primary antibody	anti-CD206/MRC1 rabbit mAb	Rabbit monoclonal antibody	E2L9N	91992T/91992S	Shanghai Universal Biotech Co., Ltd.	IHC	Used for CD206/MRC1 immunohistochemistry in local tissue sections
Secondary detection reagent	SignalStain® Boost IHC Detection Reagent (HRP, Rabbit)	HRP-labeled goat anti-rabbit IgG IHC detection reagent	NA	8114/8114S	Shanghai Universal Biotech Co., Ltd.	IHC detection	Secondary detection reagent for rabbit primary antibodies
Chromogenic reagent	SignalStain® DAB Substrate Kit	DAB substrate kit	NA	8059/8059P/8059S	Shanghai Universal Biotech Co., Ltd.	DAB chromogenic detection	Used for HRP-based chromogenic visualization

Note: This table summarizes primary antibodies, secondary detection reagent, and DAB substrate information used for local-tissue IHC analyses. PLK1, polo-like kinase 1; FOXM1, forkhead box M1; CD, cluster of differentiation; CD206/MRC1, cluster of differentiation 206/mannose receptor C-type 1; mAb, monoclonal antibody; IHC, immunohistochemistry; HRP, horseradish peroxidase; IgG, immunoglobulin G; DAB, 3,3′-diaminobenzidine; NA, not applicable. All IHC antibodies and detection reagents were used according to the manufacturers' instructions.
